# Association of Vitamin D Levels, Race/Ethnicity, and Clinical Characteristics With COVID-19 Test Results

**DOI:** 10.1001/jamanetworkopen.2021.4117

**Published:** 2021-03-19

**Authors:** David O. Meltzer, Thomas J. Best, Hui Zhang, Tamara Vokes, Vineet M. Arora, Julian Solway

**Affiliations:** 1 The University of Chicago, Chicago, Illinois

## Abstract

**Importance:**

Deficient (ie, <20 ng/mL) or insufficient (ie, 20 to <30 ng/mL) 25-hydroxyvitamin D (also known as *calcifediol*) levels are more common in Black individuals than White individuals and are associated with increased coronavirus disease 2019 (COVID-19) risk. Whether COVID-19 risk is associated with differences in vitamin D levels of 30 ng/mL or greater is not known.

**Objective:**

To examine whether COVID-19 test results are associated with differences in vitamin D levels of 30 ng/mL or greater, including for White individuals and for Black individuals.

**Design, Setting, and Participants:**

This retrospective cohort study was conducted at an academic medical center in Chicago, Illinois. Participants included individuals with data on vitamin D level within 365 days before COVID-19 testing, which was conducted from March 3 to December 30, 2020. Data were analyzed from September 11, 2020, to February 5, 2021.

**Exposures:**

The last vitamin D level before COVID-19 testing was categorized as less than 20 ng/mL (ie, deficient), 20 to less than 30 ng/mL (ie, insufficient), 30 to less than 40 ng/mL, or 40 ng/mL or greater. Treatment was defined by vitamin D type and dose 14 days before COVID-19 testing and treatment changes after last vitamin D level.

**Main Outcomes and Measures:**

The main outcome was a positive result for COVID-19 in polymerase chain reaction testing. Multivariable analyses tested whether previously measured vitamin D level was associated with having test results positive for COVID-19 in White individuals and in Black individuals, controlling for months and treatment changes since the vitamin D level was measured, as well as demographic characteristics and comorbidity indicators.

**Results:**

A total of 4638 individuals (mean [SD] age 52.8 [19.5] years; 3205 [69%] women) had data for a vitamin D level within 1 year before COVID-19 testing, including 2288 (49%) Black individuals, 1999 (43%) White individuals, and 351 individuals (8%) who were another race/ethnicity (eg, Asian, Mideast Indian, >1 race). Stratified by vitamin D level, 1251 individuals (27%) had less than 20 ng/mL, 1267 individuals (27%) had 20 to less than 30 ng/mL, 1023 individuals (22%) had 30 to less than 40 ng/mL, and 1097 individuals (24%) had 40 ng/mL or greater. Lower vitamin D levels were more common in Black individuals (<20 ng/mL: 829 of 2288 Black individuals [36%]) than White individuals (<20 ng/mL: 315 of 1999 White individuals [16%]). A total of 333 individuals (7%) had test results positive for COVID-19, including 102 White individuals (5%) and 211 Black individuals (9%). Multivariate analysis controlling for time since last vitamin D level measurement was used to estimate the outcomes associated with levels 14 days before COVID-19 testing. A positive test result for COVID-19 was not significantly associated with vitamin D levels in White individuals but was associated with vitamin D levels in Black individuals (compared with ≥40 ng/mL: <20 ng/mL incidence rate ratio [IRR], 2.55 [95% CI, 1.26-5.15]; *P* = .009; 20 to <30 ng/mL IRR, 1.69 [95% CI, 0.75-3.84]; *P* = .21; 30 to <40 ng/mL IRR, 2.64 [95% CI, 1.24-5.66]; *P* = .01). Stratified by vitamin D level, estimated COVID-19 positivity rates in Black individuals were 9.72% (95% CI, 6.74%-13.41%) for individuals with a vitamin D level less than 20 ng/mL, 6.47% (95% CI, 3.33%-10.28%) for individuals with a vitamin D level of 20 to less than 30 ng/mL, 10.10% (95% CI, 6.00%-15.47%) for individuals with a vitamin D level of 30 to less than 40 ng/mL, and 3.82% (95% CI, 1.78%-6.68%) for individuals with a vitamin D level of 40 ng/mL or higher. Multivariate analysis in individuals with a vitamin D level of 30 ng/mL or greater found that the IRR of a positive COVID-19 test result was 0.97 (95% CI, 0.94-0.99; *P* = .008) per 1-ng/mL increase in vitamin D overall and 0.95 (95% CI, 0.91-0.98; *P* = .003) per 1-ng/mL increase in vitamin D in Black individuals.

**Conclusions and Relevance:**

In this single-center retrospective cohort study, COVID-19 risk increased among Black individuals with vitamin D level less than 40 ng/mL compared with those with 40 ng/mL or greater and decreased with increasing levels among individuals with levels greater than 30 ng/mL. No significant associations were noted for White individuals. Randomized clinical trials should examine whether increasing vitamin D level to greater than 40 ng/mL affects COVID-19 risk.

## Key Points

**Question:**

Are differences in vitamin D levels greater than levels traditionally considered sufficient (30 ng/mL) associated with having test results positive for coronavirus disease 2019 (COVID-19) in White and in Black individuals?

**Findings:**

In this cohort study of 4638 individuals with a measured vitamin D level in the year before undergoing COVID-19 testing, the risk of having positive results in Black individuals was 2.64-fold greater with a vitamin D level of 30 to 39.9 ng/mL than a level of 40 ng/mL or greater and decreased by 5% per 1-ng/mL increase in level among individuals with a level of 30 ng/mL or greater. There were no statistically significant associations of vitamin D levels with COVID-19 positivity rates in White individuals.

**Meaning:**

These findings suggest that randomized clinical trials to determine whether increasing vitamin D levels to greater than 30 to 40 ng/mL affect COVID-19 risk are warranted, especially in Black individuals.

## Teaser

This cohort study examines the associations of vitamin D levels, race, and other characteristics with positive COVID-19 test results.

## Introduction

Vitamin D has diverse physiological effects, including on calcium regulation, bone density, and immune function. Deficient levels, typically defined as 25-hydroxyvitamin D (also known as *calcifediol*) level less than 20 ng/mL (to convert to nanomoles per liter, multiply by 2.496), are common, especially in Black individuals, and some expert opinions define sufficient levels as 30 ng/mL or greater or 40 ng/mL or greater.^1,2,3^ Definitions of vitamin D deficiency and supplementation guidelines have been largely informed by effects on bone health from mostly White populations,^4^ and Black individuals have been reported to preserve bone health even with lower vitamin D levels,^5^ calling into question the notion that functional vitamin D deficiency is more common in Black individuals than White individuals. However, vitamin D is also important for immune function, and a meta-analysis of randomized clinical trials using daily or weekly dosing^6^ has found vitamin D supplementation was associated with substantially decreased viral respiratory infections, especially in individuals who were deficient in vitamin D, but also individuals without a deficient level. There is no evidence that immune function is better preserved with low vitamin D levels in Black individuals than in White individuals. This is especially important currently because deficient vitamin D levels have been associated with increased COVID-19 incidence and worse outcomes, especially in Black, Hispanic, and other non-White populations, who have also borne a disproportionate burden of COVID-19.^7,8,9,10,11,12,13,14^ Evidence on whether vitamin D levels above the deficient range are associated with COVID-19 risk, and whether such associations differ between White individuals and individuals of other races, could inform the design of randomized clinical trials to test whether vitamin D supplementation reduces COVID-19 risk^15^ and clinical decision-making before completion of such trials.^16^

We used data from the electronic health record at the University of Chicago Medicine (UCM) in Chicago, Illinois, to examine whether the likelihood of a positive COVID-19 test was associated a person’s most recent vitamin D level within 365 days before COVID-19 testing, and whether such associations were present for White individuals and for Black individuals. Because more distant level may be less informative of level at the time of COVID-19 testing, analyses controlled for the timing of the most recent measurement and changes in vitamin D treatment after that level and before COVID-19 testing.

## Methods

This cohort study was approved by the University of Chicago Biological Sciences Division institutional review board, which granted a waiver of participant consent because the data were deidentified except for elements of dates, and the institutional review board determined that the risk to the privacy of participants was minimal. This study followed the Strengthening the Reporting of Observational Studies in Epidemiology (STROBE) reporting guideline for cohort studies.

### Participants

We obtained data for all 106 635 individuals tested for COVID-19 using polymerase chain reaction testing at UCM from March 3, 2020, to December 30, 2020. This sample included approximately 4314 persons tested for COVID-19 at UCM from March 3, 2020 to April 10, 2020, on whom we reported in a prior study of vitamin D deficiency and COVID-19 risk.^10^ We obtained electronic health record data for demographic, comorbidity, laboratory, and medication information within 365 days before participants’ first COVID-19 test. Vitamin D levels and treatments within 14 days of COVID-19 testing were excluded from analyses to avoid confounding by potential early manifestations of COVID-19, for example, presenting for care with symptoms that could lead to vitamin D testing and treatment. A total of 491 individuals were excluded because the only measured vitamin D levels or treatments were within 14 days of COVID-19 testing.

### Measurements

All variables were obtained from the UCM electronic health record, Epic (Epic Systems). COVID-19 status was determined by the Centers for Disease Control and Prevention^17^ or Viacor^18^ polymerase chain reaction(PCR) test used until in-house testing with the Cobas SARS-CoV-2 RT-PCR (Roche) began March 15, 2020.^19^ Testing initially focused on individuals presenting with potential COVID-19 symptoms admitted to the hospital or health care workers with COVID-19 symptoms and exposure and was gradually broadened. Individuals’ most recent vitamin D level within 365 days before their first COVID-19 test was used to classify their level as less than 20 ng/mL (ie, deficient), 20 to less than 30 ng/mL (ie, insufficient), 30 to less than 40 ng/mL, or 40 ng/mL or greater. An additional analysis controlled for months from most recent vitamin D level measurement to 14 days before COVID-19 test result. The vitamin D dosing regimen was defined by the type of vitamin D (D_2_, D_3_, or calcitriol) and mean daily dose of vitamin D_3_. Changes in individuals’ recorded vitamin D treatment between the date of their most recent vitamin D level measurement and 14 days before COVID-19 testing were categorized as increased, unchanged, or decreased based on movement between the following regimens, from highest to lowest: 4001 IU/d or more vitamin D_3_, 2001 to 4000 IU/d vitamin D_3_, 1001 to 2000 IU/d vitamin D_3_, D_2_, 1 to 1000 IU/d vitamin D_3_ or a daily multivitamin, and no vitamin D.

Age, sex, race (classified as White, Black, or other), and Hispanic/Latino ethnicity were collected based on participant self-selection among institutionally selected options. We used the most recent data from 365 to 14 days before COVID-19 testing to calculate body mass index. The Elixhauser comorbidity clusters potentially related to COVID-19 or vitamin D status were calculated using *International Statistical Classification of Diseases, Tenth Revision, Clinical Modification (ICD-10-CM)*^20^ codes during 2 years, including hypertension, immunosuppression, diabetes, renal failure, chronic pulmonary disease, depression, liver disease, any psychosis, any pulmonary circulation disorder, and dementia.^21^

### Statistical Analysis

Descriptive statistics were reviewed and compared using χ^2^ test or analysis of variance, including rates of positive test results for COVID-19, stratified by vitamin D level. Multivariable analyses were performed for the full population and separately for White and Black individuals. A multivariable generalized linear model with Poisson residuals and logit link function^22,23^ was estimated with the aforementioned covariates except months from last measured vitamin D level to 14 days before COVID-19 testing. Another model controlled for months from the last measured vitamin D level to 14 days before COVID-19 testing and interaction of the months variable with the last measured vitamin D level. This specification allows the association of vitamin D level with the outcome to vary by time since COVID-19 testing. The coefficient on the level variable (main term) indicates the estimated association with the vitamin D level 14 days before COVID-19 testing. Estimated probabilities of a positive COVID-19 test result were compared across level categories using bootstrapped bias-corrected 95% CIs to adjust for potential nonnormality in the distribution of the estimated probabilities. Additional analyses examined individuals with a vitamin D level of 30 ng/mL or greater, including analysis of vitamin D as a continuous variable. Multivariable model selection was informed by information criteria (Akaike or Bayesian), examination of residuals vs estimated values, and goodness-of-link and Hosmer-Lemeshow tests,^24,25^ resulting in inclusion of interactions of age with month of testing. We also examined rates of positive test results stratified by most recent vitamin D level and recorded vitamin D_3_ dosing (including none) 14 days before COVID-19 testing after excluding individuals using vitamin D_2_ or 1,25-dihydroxyvitamin D supplements at that time. Sensitivity analyses repeated multivariable analyses in persons with last vitamin D level measurement within 90 days of COVID-19 testing, stratified by whether persons were recorded as using vitamin D supplements at 14 days before COVID testing, and combining all non-White individuals, including Black, Mideast Indian, Asian, and other or multiple races. Statistical analysis was performed in Stata statistical software (StataCorp). *P* values were 2-sided, and statistical significance was set at *P* = .05. Data were analyzed from September 11, 2020, to February 5, 2021.

## Results

### Characteristics of the Sample

Of 106 635 individuals tested for COVID-19 at UCM from March 3, 2020, to December 30, 2020, 4941 individuals had data on vitamin D level from within 365 days before COVID-19 testing, and 4638 individuals had complete data and were included in our analytic sample. The mean (SD) age was 52.8 (19.5) years, 3205 (69%) were women, and 1999 individuals (43%) were White, 2288 individuals (49%) were Black, and 351 (8%) individuals were another race (Table 1). Mean (SD) time since last measured vitamin D level was 167 (103) days and did not vary significantly by vitamin D level. The most recent vitamin D level was less than 20 ng/mL for 1251 individuals (27%), 20 to less than 30 ng/mL for 1267 individuals (27%), 30 to less than 40 ng/mL for 1023 individuals (22%), and greater than 40 ng/mL for 1097 individuals (24%).

**Table 1.** Characteristics of the Sample

Characteristic	No. (%)					χ^2^ test for independence, *P* value
	Full sample (N = 4638)	Most recent vitamin D level from 14 to 365 d before COVID-19 test order, ng/mL				
		≥40 (n = 1097)	30-39 (n = 1023)	20-29 (n = 1267)	<20 (n = 1251)	
Age, y						
Mean (SD)	52.8 (19.5)	57.9 (19.9)	55.6 (19.1)	50.8 (18.9)	48.1 (18.6)	<.001^a^
<50	1908 (41)	311 (28)	346 (34)	586 (46)	665 (53)	<.001
50-64	1236 (27)	287 (26)	292 (29)	347 (27)	310 (25)	
≥65	1494 (32)	499 (45)	385 (38)	334 (26)	276 (22)	
Sex						
Women	3205 (69)	791 (72)	703 (69)	863 (68)	848 (68)	.02
Men	1433 (31)	306 (28)	320 (31)	404 (32)	403 (32)	
Race						
White	1999 (43)	602 (55)	506 (49)	576 (45)	315 (25)	<.001
Black	2288 (49)	432 (39)	441 (43)	586 (46)	829 (66)	
Other^b^	351 (8)	63 (6)	76 (7)	105 (8)	107 (9)	
Ethnicity						
Hispanic	307 (7)	64 (6)	53 (5)	97 (8)	93 (7)	.04
Non-Hispanic	4331 (93)	1033 (94)	970 (95)	1170 (92)	1158 (93)	
Employed at University of Chicago						
Yes	557 (12)	110 (10)	116 (11)	185 (15)	146 (12)	.005
No	4081 (88)	987 (90)	907 (89)	1082 (85)	1105 (88)	
Most recent vitamin D level before COVID-19 test, mean (SD), ng/mL	30.3 (15.9)	52.6 (12.4)	34.2 (2.9)	24.5 (2.9)	13.4 (3.8)	<.001^a^
Time since most recent vitamin D level measurement, d						
Mean (SD)	167 (103)	161 (105)	166 (105)	170 (102)	169 (101)	.17^a^
Median (IQR)	154 (180)	147 (179)	157 (188)	160 (181)	156 (172)	.24
Vitamin D dosage change after most recent vitamin D evaluation^b^						
No change	3968 (86)	988 (90)	921 (90)	1092 (86)	967 (77)	<.001
Increased	477 (10)	64 (6)	69 (7)	125 (10)	219 (18)	
Decreased	193 (4)	45 (4)	33 (3)	50 (4)	65 (5)	
Time of COVID-19 test						
March	323 (7)	53 (5)	57 (6)	93 (7)	120 (10)	<.001
April	431 (9)	84 (8)	76 (7)	119 (9)	152 (12)	
May	545 (12)	101 (9)	124 (12)	144 (11)	176 (14)	
June	624 (13)	159 (14)	131 (13)	160 (13)	174 (14)	
July	607 (13)	131 (12)	135 (13)	175 (14)	166 (13)	
August	453 (10)	119 (11)	107 (10)	121 (10)	106 (8)	
September	362 (8)	100 (9)	82 (8)	96 (8)	84 (7)	
October	483 (10)	135 (12)	117 (11)	130 (10)	101 (8)	
November	479 (10)	125 (11)	116 (11)	138 (11)	100 (8)	
December	331 (7)	90 (8)	78 (8)	91 (7)	72 (6)	
Comorbidity						
Hypertension	2367 (51)	583 (53)	541 (53)	598 (47)	645 (52)	.01
Comorbidity with immunosuppression	1169 (25)	317 (29)	243 (24)	305 (24)	304 (24)	.01
Diabetes	1156 (25)	270 (25)	271 (26)	289 (23)	326 (26)	.15
Renal failure	938 (20)	241 (22)	210 (21)	227 (18)	260 (21)	.09
Chronic pulmonary disease	922 (20)	205 (19)	206 (20)	230 (18)	281 (22)	.03
Depression	796 (17)	168 (15)	158 (15)	210 (17)	260 (21)	<.001
Liver disease	614 (13)	158 (14)	134 (13)	158 (12)	164 (13)	.58
Psychosis	298 (6)	79 (7)	57 (6)	71 (6)	91 (7)	.15
Pulmonary circulation disorder	245 (5)	56 (5)	46 (4)	63 (5)	80 (6)	.20
Dementia	104 (2)	23 (2)	26 (3)	21 (2)	34 (3)	.29
BMI						
Mean (SD)	29.4 (8.3)	27.8 (7.4)	29.3 (7.8)	29.9 (8.8)	30.2 (8.9)	<.001^a^
≥30	1869 (40)	357 (33)	400 (39)	537 (42)	575 (46)	<.001
Most recent active vitamin D treatment before COVID-19 test, IU/d D_3_^c^						<.001
None	3338 (72)	788 (72)	733 (72)	917 (72)	900 (72)	.98
1-1000 or multivitamin	616 (13)	162 (15)	167 (16)	171 (13)	116 (9)	<.001
D_2_	346 (7)	44 (4)	56 (5)	84 (7)	162 (13)	<.001
1001-2000	173 (4)	41 (4)	39 (4)	57 (4)	36 (3)	.20
2001-4000	28 (1)	10 (1)	7 (1)	7 (1)	4 (<1)	.31
≥4001	103 (2)	45 (4)	15 (1)	23 (2)	20 (2)	<.001
Calcitriol	34 (1)	7 (1)	6 (1)	8 (1)	13 (1)	.52
Test result positive for COVID-19	333 (7)	61 (6)	78 (8)	76 (6)	118 (9)	<.001

Abbreviations: BMI, body mass index (calculated as weight in kilograms divided by height in meters squared); COVID-19, coronavirus disease 2019.

Conversion factor: To convert vitamin D to nanomoles per liter, multiply by 2.496.

^a^ *P* value determined via analysis of variance.

^b^ Other includes 184 Asian/Mideast Indian individuals, 138 individuals with more than 1 race, 21 American Indian or Alaska Native individuals, and 8 Native Hawaiian or Other Pacific Islander individuals.

^c^ Vitamin D dose was rank-ordered from highest to lowest as calcitriol, 4001 IU/d or greater D_3_, 2001 to 4000 IU/d D_3_, 1001 to 2000 IU/d D_3_, D_2_, > 1 to 1000 IU/d D_3_ or multivitamin, and no vitamin D.

Table 1 also provides descriptive statistics for demographic and comorbidity measures, vitamin D deficiency, treatments, and rates of positive COVID-19 test results stratified by categories of vitamin D level. Vitamin D levels were lower among individuals who were younger, male, Black, Hispanic, University of Chicago employees, tested for COVID-19 earlier in the calendar year, or obese and among individuals who had a treatment dose increase after the most recent vitamin D level measurement, chronic pulmonary disease, or depression. Recorded vitamin D treatments 14 days before COVID-19 testing were similar across vitamin D categories, except that individuals who were deficient in vitamin D were recorded more frequently using vitamin D_2_ and less frequently using vitamin D_3_ at a dosage of 1000 IU/d or less, and individuals with vitamin D level 40 ng/mL or greater were more often recorded as using 4001 IU/d or more vitamin D_3_.

### Follow-up and Outcomes

Overall, 333 individuals (7%) had test results positive for COVID-19, including 118 of 1251 individuals (9%) with vitamin D level less than 20 ng/mL (*P* < .001 vs individuals with vitamin D level ≥40 ng/mL), 76 of 1267 individuals (6%) with a vitamin D level of 20 to less than 30 ng/mL (*P* = .65 vs individuals with vitamin D level ≥40 ng/mL), 78 of 1023 individuals (8%) with a vitamin D level of 30 to less than 40 ng/mL (*P* = .06 vs individuals with vitamin D level ≥40 ng/mL), and 61 of 1097 individuals (6%) with vitamin D level 40 ng/mL or higher (*P* < .001 overall). Among Black individuals, 211 (9%) had positive COVID-19 test results, compared with 102 White individuals (5%) (*P* < .001). The eFigure in the Supplement reports COVID-19 positivity rates by race and vitamin D level.

Table 2 and Table 3 report the multivariable analysis of COVID-19 testing results for the full sample and in Black individuals. In model 1, the model not controlling for time since the most recent vitamin D level measurement for the full sample, the incidence rate ratio (IRR) of having test results positive for COVID-19 was 1.41 (95% CI, 1.04-1.92; *P* = .03) for individuals with deficient vitamin D level, 0.95 (95% CI, 0.69-1.32; *P* = .78) for individuals with insufficient vitamin D level, and 1.27 (95% CI, 0.93-1.75; *P* = .14) for individuals with a vitamin D level of 20 to less than 30 ng/mL, compared with individuals with a vitamin D level of 40 ng/mL or greater. In model 2, the model controlling for time since last vitamin D level to estimate the association of vitamin D levels 14 days before COVID-19 testing with COVID-19 test results, the IRR of positive results for COVID-19 was 1.53 (95% CI, 0.91-2.56; *P* = .11) for individuals with vitamin D level less than 20 ng/mL, 1.21 (95% CI, 0.68-2.13; *P* = .52) for individuals with a vitamin D level of 20 to less than 30 ng/mL, and 1.42 (95% CI, 0.82-2.48; *P* = .21) for individuals with a vitamin D level of 30 to less than 40 ng/mL compared with individuals with a vitamin D level of 40 ng/mL or greater. Time since most recent measurement of vitamin D level was not associated with the risk of having positive results for COVID-19. In the models limited to individuals with a vitamin D level of 30 ng/mL or greater, a vitamin D level of 40 ng/mL or greater was not significantly associated with increased risk (model 3), but a 1-ng/mL increase in vitamin D level was associated with a 3% decrease in risk of positive COVID-19 test results (model 4) (IRR, 0.97 [95% CI, 0.94-0.99]; *P* = .008). Having a positive test result for COVID-19 was also associated with dementia, and age interacted with testing in March or April in all models, Black race and testing in April or November interacted in models with all individuals, and employment at the University of Chicago and age interacted with testing in November in models limited to individuals with a vitamin D level of 30 ng/mL or greater. Having negative test results for COVID-19 was associated with immunosuppressed conditions and testing in June through September in all models, female sex in models with all individuals, and age in models limited to individuals with a vitamin D level of 30 ng/mL or greater.

**Table 2.** Multivariable Association of Vitamin D Levels and Treatment With Test Results Positive for COVID-19

Characteristic	Models 1 and 2, No. (%) (n = 4638)	Model 1^a^		Model 2 ^b^		Models 3 and 4, No. (%) (n = 2120)	Model 3^c^		Model 4^d^	
		IRR (95% CI)	*P* value	IRR (95% CI)	*P* value		IRR (95% CI)	*P* value	IRR (95% CI)	*P* value
Sex										
Men	1433 (31)	1 [Reference]	NA	1 [Reference]	NA	626 (30)	1 [Reference]	NA	1 [Reference]	NA
Women	3205 (69)	0.69 (0.55-0.86)	.001	0.69 (0.55-0.86)	.001	1494 (70)	0.84 (0.58-1.22)	.37	0.85 (0.59-1.24)	.41
Race										
White	1999 (43)	1 [Reference]	NA	1 [Reference]	NA	1108 (52)	1 [Reference]	NA	1 [Reference]	NA
Black	2288 (49)	1.49 (1.15-1.92)	.003	1.48 (1.14-1.91)	.003	873 (41)	1.28 (0.88-1.86)	.20	1.28 (0.87-1.87)	.20
Other	351 (8)	0.84 (0.52-1.33)	.45	0.83 (0.52-1.32)	.42	139 (7)	1.01 (0.46-2.18)	.99	0.96 (0.44-2.06)	.91
Ethnicity										
Non-Hispanic	4331 (93)	1 [Reference]	NA	1 [Reference]	NA	2003 (94)	1 [Reference]	NA	1 [Reference]	NA
Hispanic	307 (7)	1.41 (0.91-2.19)	.12	1.42 (0.92-2.19)	.12	117 (6)	1.36 (0.66-2.79)	.41	1.39 (0.67-2.88)	.37
University of Chicago employee										
No	4081 (88)	[Reference]	NA	[Reference]	NA	1894 (89)	[Reference]	NA	[Reference]	NA
Yes	557 (12)	1.31 (0.96-1.79)	.09	1.30 (0.95-1.78)	.10	226 (11)	1.65 (1.05-2.59)	.03	1.66 (1.06-2.61)	.03
Most recent vitamin D level before COVID-19 test, ng/mL										
Mean (SD)	30.3 (15.9)	NA	NA	NA	NA	43.7 (13.0)	NA	NA	0.97 (0.94-0.99)	.008
≥40	1097 (24)	1 [Reference]	NA	1 [Reference]	NA	1097 (52)	1 [Reference]	NA	1 [Reference]	NA
30-39	1023 (22)	1.27 (0.93-1.75)	.14	1.42 (0.82-2.48)	.21	1023 (48)	1.35 (0.78-2.34)	.28	NA	NA
20-29	1267 (27)	0.95 (0.69-1.32)	.78	1.21 (0.68-2.13)	.52	NA	NA	NA	NA	NA
<20	1251 (27)	1.41 (1.04-1.92)	.03	1.53 (0.91-2.56)	.11	NA	NA	NA	NA	NA
Time since most recent vitamin D level measurement, mean (SD), mo	5.5 (3.4)	NA	NA	1.04 (0.98-1.11)	.22	5.4 (3.5)	1.05 (0.98-1.12)	.13	0.93 (0.79-1.10)	.41
Interactions between vitamin D level and time since most recent vitamin D measurement										
30-39	NA	NA	NA	0.98 (0.90-1.07)	.61	NA	0.98 (0.89-1.07)	.59	1.00 (1.00-1.01)	.19
20-29	NA	NA	NA	0.95 (0.87-1.04)	.31	NA	NA	NA		
<20	NA	NA	NA	0.98 (0.91-1.07)	.70	NA	NA	NA		
Vitamin D dosage change after most recent vitamin D measurement level										
Increased	477 (10)	0.94 (0.68-1.29)	.70	0.92 (0.67-1.27)	.63	133 (6)	0.75 (0.41-1.40)	.37	0.74 (0.40-1.38)	.35
Decreased	193 (4)	1.21 (0.74-1.98)	.45	1.19 (0.72-1.96)	.50	78 (4)	0.30 (0.08-1.15)	.08	0.33 (0.09-1.29)	.11
Demeaned age	NA	0.98 (0.97-1.00)	.07	0.98 (0.97-1.00)	.07	NA	0.97 (0.95-1.00)	.03	0.97 (0.95-1.00)	.03
Time of COVID-19 test										
December	331 (7)	1 [Reference]	NA	1 [Reference]	NA	168 (8)	1 [Reference]	NA	1 [Reference]	NA
March	323 (7)	1.41 (0.87-2.28)	.16	1.41 (0.87-2.28)	.16	110 (5)	0.95 (0.38-2.38)	.91	0.92 (0.37-2.30)	.86
April	431 (9)	1.55 (1.01-2.40)	.05	1.55 (1.01-2.40)	.05	160 (8)	1.69 (0.83-3.44)	.14	1.70 (0.83-3.48)	.15
May	545 (12)	0.68 (0.42-1.12)	.13	0.68 (0.42-1.12)	.13	225 (11)	0.91 (0.43-1.94)	.82	0.88 (0.41-1.87)	.73
June	624 (13)	0.21 (0.11-0.42)	<.001	0.21 (0.11-0.41)	<.001	290 (14)	0.20 (0.07-0.58)	.003	0.20 (0.07-0.59)	.003
July	607 (13)	0.17 (0.08-0.37)	<.001	0.17 (0.08-0.36)	<.001	266 (13)	0.23 (0.08-0.66)	.007	0.23 (0.08-0.67)	.007
August	453 (10)	0.24 (0.11-0.53)	<.001	0.24 (0.11-0.52)	<.001	226 (11)	0.12 (0.03-0.61)	.01	0.13 (0.03-0.61)	.01
September	362 (8)	0.15 (0.05-0.40)	<.001	0.14 (0.05-0.39)	<.001	182 (9)	0.09 (0.02-0.42)	.002	0.09 (0.02-0.42)	.002
October	483 (10)	0.99 (0.62-1.60)	.98	0.99 (0.61-1.59)	.96	252 (12)	1.12 (0.55-2.28)	.75	1.10 (0.54-2.26)	.79
November	479 (10)	1.89 (1.24-2.88)	.003	1.89 (1.24-2.87)	.003	241 (11)	1.71 (0.88-3.31)	.11	1.68 (0.86-3.28)	.13
Interactions between time of COVID-19 test and demeaned age										
December	NA	1 [Reference]	NA	1 [Reference]	NA	NA	1 [Reference]	NA	1 [Reference]	NA
March	NA	1.04 (1.02-1.07)	<.001	1.04 (1.02-1.07)	<.001	NA	1.11 (1.06-1.15)	<.001	1.11 (1.06-1.15)	<.001
April	NA	1.03 (1.01-1.05)	.007	1.03 (1.01-1.05)	.008	NA	1.05 (1.02-1.08)	.003	1.05 (1.02-1.09)	.004
May	NA	1.01 (0.99-1.04)	.23	1.01 (0.99-1.04)	.24	NA	1.02 (0.98-1.05)	.33	1.02 (0.98-1.06)	.31
June	NA	0.98 (0.96-1.00)	.11	0.98 (0.96-1.00)	.09	NA	0.98 (0.95-1.02)	.34	0.98 (0.95-1.02)	.34
July	NA	0.98 (0.95-1.01)	.16	0.98 (0.95-1.01)	.16	NA	0.99 (0.95-1.02)	.45	0.99 (0.95-1.02)	.51
August	NA	0.98 (0.95-1.02)	.40	0.98 (0.95-1.02)	.40	NA	0.99 (0.93-1.05)	.74	0.99 (0.93-1.05)	.76
September	NA	0.99 (0.95-1.04)	.80	0.99 (0.95-1.04)	.80	NA	0.97 (0.94-1.00)	.05	0.97 (0.94-1.00)	.06
October	NA	1.01 (0.99-1.04)	.26	1.01 (0.99-1.04)	.26	NA	1.03 (1.00-1.06)	.06	1.03 (1.00-1.06)	.06
November	NA	1.02 (1.00-1.04)	.10	1.02 (1.00-1.04)	.09	NA	1.04 (1.01-1.07)	.01	1.04 (1.01-1.07)	.006
Comorbidity										
Hypertension	2367 (51)	1.17 (0.88-1.54)	.28	1.16 (0.88-1.54)	.28	1124 (53)	1.46 (0.96-2.24)	.08	1.46 (0.96-2.23)	.08
Comorbidity with immunosuppression	1169 (25)	0.77 (0.59-1.00)	.05	0.76 (0.58-1.00)	.05	560 (26)	0.58 (0.38-0.90)	.01	0.61 (0.39-0.94)	.03
Diabetes	1156 (25)	1.15 (0.90-1.47)	.28	1.14 (0.89-1.47)	.29	541 (26)	0.97 (0.66-1.43)	.89	0.97 (0.66-1.44)	.89
Renal failure	938 (20)	0.98 (0.74-1.29)	.87	0.98 (0.74-1.29)	.87	451 (21)	0.92 (0.61-1.38)	.67	0.92 (0.61-1.39)	.69
Chronic pulmonary disease	922 (20)	0.85 (0.64-1.11)	.23	0.84 (0.64-1.11)	.22	411 (19)	0.88 (0.58-1.34)	.55	0.91 (0.59-1.39)	.66
Depression	796 (17)	1.25 (0.96-1.62)	.09	1.23 (0.95-1.60)	.11	326 (15)	1.41 (0.94-2.10)	.10	1.38 (0.92-2.06)	.12
Liver disease	614 (13)	0.93 (0.67-1.30)	.67	0.94 (0.67-1.31)	.71	292 (14)	0.88 (0.54-1.45)	.62	0.90 (0.55-1.48)	.67
Psychosis	298 (6)	0.69 (0.44-1.09)	.11	0.69 (0.43-1.09)	.11	136 (6	0.75 (0.37-1.54)	.43	0.74 (0.36-1.53)	.42
Pulmonary circulation disorder	245 (5)	0.80 (0.49-1.32)	.39	0.80 (0.49-1.32)	.39	102 (5)	0.70 (0.27-1.84)	.47	0.69 (0.26-1.81)	.45
Dementia	104 (2)	2.12 (1.41-3.20)	<.001	2.16 (1.44-3.26)	<.001	49 (2)	1.89 (1.11-3.20)	.02	1.80 (1.05-3.10)	.03
BMI, mean (SD)	29.4 (8.3)	1.01 (1.00-1.02)	.07	1.01 (1.00-1.02)	.07	28.5 (7.6)	1.01 (0.99-1.03)	.38	1.01 (0.99-1.03)	.54

Abbreviations: BMI, body mass index (calculated as weight in kilograms divided by height in meters squared); COVID-19, Coronavirus Disease 2019; IRR, incidence rate ratio; NA, not applicable.

Conversion factor: To convert vitamin D to nanomoles per liter, multiply by 2.496.

^a^ Adjusted for all covariates. Goodness-of-link test of squared predicted value *P* = .52; Hosmer-Lemeshow goodness-of-fit decile test, *P* = .19.

^b^ Adjusted for items included in model 1 plus the interaction between most recent vitamin D level and months since measurement, defined as the number of days between an individual’s most recent vitamin D measurement and 14 days before the individual’s first COVID-19 test order, divided by (365 / 12). Goodness-of-link test of squared predicted value *P* = .59; Hosmer-Lemeshow goodness-of-fit decile test, *P* = .23.

^c^ Adjusted for items included in model 2 and restricted to individuals with vitamin D levels of 30 ng/mL or greater. Goodness-of-link test of squared predicted value *P* = .47; Hosmer-Lemeshow goodness-of-fit decile test, *P* = .23.

^d^ Adjusted for items included in model 3 but vitamin D level is treated as a continuous rather than categorical variable. Goodness-of-link test of squared predicted value *P* = .21; Hosmer-Lemeshow goodness-of-fit decile test, *P* = .51.

**Table 3.** Multivariable Association of Vitamin D Levels and Treatment With Testing Positive for COVID-19 Among Individuals Reporting Black Race

Characteristic	Models 1 and 2, No. (%) (n = 2288)	Model 1^a^		Model 2^b^		Models 3 and 4, No. (%) (n = 873)	Model 3^c^		Model 4^d^	
		IRR (95% CI)	*P* value	IRR (95% CI)	*P* value		IRR (95% CI)	*P* value	IRR (95% CI)	*P* value
Sex										
Men	621 (27)	1 [Reference]	NA	1 [Reference]	NA	220 (25)	1 [Reference]	NA	1 [Reference]	NA
Women	1667 (73)	0.70 (0.53-0.93)	.01	0.70 (0.53-0.92)	.01	653 (75)	0.85 (0.48-1.50)	.58	0.89 (0.51-1.57)	.69
Ethnicity										
Non-Hispanic	2280 (>99)	1 [Reference]	NA	1 [Reference]	NA	872 (>99)	1 [Reference]	NA	1 [Reference]	NA
Hispanic^e^	8 (<1)	NA	NA	NA	NA	1 (<1)	NA	NA	NA	NA
University of Chicago employee										
No	2081 (91)	1 [Reference]	NA	1 [Reference]	NA	810 (93)	1 [Reference]	NA	1 [Reference]	NA
Yes	207 (9)	1.19 (0.76-1.86)	.46	1.16 (0.74-1.82)	.52	63 (7)	1.24 (0.57-2.70)	.59	1.23 (0.56-2.69)	.60
Most recent vitamin D level, mean (SD), ng/mL	27.5 (16.1)					43.7 (13.5)			0.95 (0.91-0.98)	.003
≥40	432 (19)	1 [Reference]	NA	1 [Reference]	NA	432 (49)	1 [Reference]	NA	NA	NA
30-39	441 (19)	1.34 (0.88-2.05)	.18	2.64 (1.24-5.66)	.01	441 (51)	2.49 (1.17-5.28)	.02	NA	NA
20-29	586 (26)	0.97 (0.62-1.52)	.90	1.69 (0.75-3.84)	.21	NA	NA	NA	NA	NA
<20	829 (36)	1.57 (1.06-2.33)	.02	2.55 (1.26-5.15)	.009	NA	NA	NA	NA	NA
Time since most recent vitamin D level, mean (SD), mo	5.5 (3.4)	NA	NA	1.12 (1.04-1.22)	.004	5.5 (3.4)	1.14 (1.04-1.24)	.003	0.82 (0.67-1.01)	.06
Interactions between vitamin D level and time since most recent vitamin D measurement										
30-39	NA	NA	NA	0.88 (0.79-0.98)	.02	NA	0.88 (0.79-0.98)	.02	1.01 (1.00-1.01)	.01
20-29	NA	NA	NA	0.91 (0.81-1.02)	.10	NA	NA	NA		
<20	NA	NA	NA	0.92 (0.84-1.01)	.09	NA	NA	NA		
Vitamin D dosage change after most recent vitamin D measurement										
Increased	272 (12)	0.84 (0.59-1.22)	.36	0.82 (0.57-1.18)	.29	56 (6)	0.85 (0.45-1.59)	.60	0.82 (0.43-1.56)	.55
Decreased	116 (5)	0.90 (0.46-1.75)	.75	0.87 (0.45-1.70)	.69	46 (5)	0.44 (0.12-1.67)	.23	0.49 (0.12-1.97)	.31
Demeaned age	NA	1.00 (0.98-1.02)	.74	1.00 (0.98-1.02)	.78	NA	0.98 (0.95-1.01)	.12	0.98 (0.95-1.01)	.12
Time period of COVID-19 test										
December	144 (6)	1 [Reference]	NA	1 [Reference]	NA	65 (7)	1 [Reference]	NA	1 [Reference]	NA
March	191 (8)	1.90 (0.99-3.62)	.05	1.88 (0.99-3.60)	.06	51 (6)	2.34 (0.76-7.25)	.14	2.26 (0.72-7.06)	.16
April	272 (12)	2.30 (1.28-4.14)	.005	2.30 (1.28-4.12)	.005	94 (11)	3.13 (1.17-8.39)	.02	3.08 (1.13-8.38)	.03
May	292 (13)	0.89 (0.46-1.72)	.72	0.88 (0.45-1.72)	.71	99 (11)	1.08 (0.33-3.48)	.90	1.04 (0.32-3.36)	.95
June	322 (14)	0.22 (0.09-0.54)	<.001	0.22 (0.09-0.53)	<.001	126 (14)	0.09 (0.01-0.74)	.03	0.09 (0.01-0.75)	.03
July	285 (12)	0.13 (0.04-0.44)	.001	0.13 (0.04-0.43)	<.001	111 (13)	0.08 (0.01-0.69)	.02	0.08 (0.01-0.71)	.02
August	201 (9)	0.27 (0.10-0.75)	.01	0.27 (0.10-0.74)	.01	86 (10)	0.36 (0.06-2.00)	.24	0.36 (0.07-1.99)	.24
September	165 (7)	0.19 (0.06-0.67)	.01	0.19 (0.05-0.65)	.009	70 (8)	0.13 (0.02-1.14)	.07	0.13 (0.02-1.13)	.06
October	193 (8)	0.78 (0.37-1.68)	.53	0.77 (0.36-1.65)	.50	79 (9)	0.79 (0.23-2.79)	.72	0.78 (0.22-2.73)	.70
November	223 (10)	2.08 (1.15-3.77)	.02	2.08 (1.15-3.76)	.02	92 (11)	2.26 (0.82-6.20)	.11	2.20 (0.79-6.10)	.13
Interactions between time of COVID-19 test and demeaned age										
December	NA	1 [Reference]	NA	1 [Reference]	NA	NA	1 [Reference]	NA	1 [Reference]	NA
March	NA	1.04 (1.01-1.07)	.01	1.04 (1.01-1.07)	.01	NA	1.09 (1.04-1.15)	<.001	1.09 (1.04-1.15)	<.001
April	NA	1.02 (0.99-1.04)	.18	1.01 (0.99-1.04)	.24	NA	1.04 (1.00-1.08)	.04	1.04 (1.00-1.08)	.05
May	NA	1.00 (0.97-1.03)	.98	1.00 (0.97-1.03)	.97	NA	1.01 (0.94-1.07)	.86	1.01 (0.94-1.07)	.88
June	NA	0.97 (0.94-1.00)	.05	0.97 (0.94-1.00)	.03	NA	0.98 (0.96-1.01)	.20	0.98 (0.96-1.01)	.25
July	NA	0.96 (0.91-1.00)	.05	0.95 (0.91-1.00)	.04	NA	0.94 (0.89-1.00)	.04	0.95 (0.90-1.00)	.05
August	NA	1.00 (0.93-1.07)	.99	1.00 (0.93-1.07)	.98	NA	0.98 (0.93-1.02)	.32	0.98 (0.93-1.02)	.36
September	NA	1.01 (0.95-1.07)	.78	1.01 (0.95-1.07)	.80	NA	0.97 (0.94-1.00)	.05	0.97 (0.93-1.00)	.07
October	NA	0.98 (0.94-1.01)	.18	0.98 (0.94-1.01)	.20	NA	1.01 (0.96-1.06)	.77	1.01 (0.96-1.06)	.72
November	NA	1.01 (0.98-1.03)	.57	1.01 (0.98-1.03)	.53	NA	1.04 (1.00-1.08)	.07	1.04 (1.00-1.08)	.06
Comorbidity										
Hypertension	1572 (69)	1.11 (0.78-1.57)	.56	1.09 (0.77-1.54)	.63	664 (76)	1.60 (0.83-3.08)	.16	1.61 (0.84-3.08)	.15
Comorbidity with immunosuppression	604 (26)	0.72 (0.51-1.00)	.05	0.70 (0.50-0.98)	.04	247 (28)	0.44 (0.24-0.80)	.007	0.45 (0.25-0.83)	.01
Diabetes	764 (33)	1.00 (0.75-1.34)	.98	1.00 (0.75-1.33)	.97	332 (38)	0.71 (0.45-1.12)	.15	0.71 (0.45-1.12)	.14
Renal failure	693 (30)	1.00 (0.73-1.37)	.98	1.01 (0.74-1.38)	.95	318 (36)	0.87 (0.53-1.44)	.60	0.88 (0.53-1.44)	.60
Chronic pulmonary disease	614 (27)	0.91 (0.67-1.25)	.57	0.92 (0.68-1.26)	.62	241 (28)	1.19 (0.73-1.94)	.48	1.26 (0.77-2.07)	.35
Depression	454 (20)	1.43 (1.05-1.94)	.02	1.40 (1.03-1.91)	.03	152 (17)	2.07 (1.30-3.28)	.002	1.98 (1.25-3.15)	.004
Liver disease	305 (13)	0.92 (0.61-1.39)	.68	0.91 (0.60-1.37)	.66	125 (14)	1.05 (0.58-1.90)	.87	1.09 (0.59-1.99)	.78
Psychosis	185 (8)	0.59 (0.34-1.04)	.07	0.58 (0.33-1.02)	.06	72 (8)	0.62 (0.26-1.45)	.27	0.62 (0.26-1.45)	.27
Pulmonary circulation disorder	158 (7)	0.62 (0.33-1.18)	.15	0.61 (0.32-1.15)	.12	53 (6)	0.20 (0.04-0.97)	.05	0.21 (0.04-1.04)	.06
Dementia	92 (4)	1.84 (1.20-2.83)	.005	1.98 (1.29-3.03)	.002	43 (5)	2.19 (1.15-4.16)	.02	2.15 (1.10-4.21)	.03
BMI, mean (SD)	30.9 (9.2)	1.01 (1.00-1.02)	.09	1.01 (1.00-1.02)	.08	30.1 (8.4)	1.01 (0.98-1.04)	.42	1.01 (0.98-1.03)	.60

Abbreviations: BMI, body mass index (calculated as weight in kilograms divided by height in meters squared); COVID-19, Coronavirus Disease 2019; IRR, incidence rate ratio; NA, not applicable.

Conversion factor: To convert vitamin D to nanomoles per liter, multiply by 2.496.

^a^ Adjusted for all covariates. Goodness-of-link test of squared predicted value *P* = .42; Hosmer-Lemeshow goodness-of-fit decile test, *P* = .72.

^b^ Adjusted for items included in model 1 plus the interaction between most recent vitamin D level and months since measurement, defined as the number of days between an individual’s most recent vitamin D measurement and 14 days before the individual’s first COVID-19 test order, divided by (365 / 12). Goodness-of-link test of squared predicted value *P* = .55; Hosmer-Lemeshow goodness-of-fit decile test, *P* = .89.

^c^ Adjusted for items included in model 2 and restricted to individuals with vitamin D levels of 30 ng/mL or greater. Goodness-of-link test of squared predicted value *P* = .24; Hosmer-Lemeshow goodness-of-fit decile test, *P* = .97.

^d^ Adjusted for items included in model 3 but vitamin D level is treated as a continuous rather than categorical variable. Goodness-of-link test of squared predicted value *P* = .08; Hosmer-Lemeshow goodness-of-fit decile test, *P* = .82.

^e^ Stata did not produce a reliable coefficient estimate for the ethnicity indicator because 0 of the 8 Hispanic persons in this subsample tested positive for COVID-19, so we omitted the ethnicity covariate as indicated.

For Black individuals, the IRR of test results positive for COVID-19 in the model not controlling for time since the most recent measurement of vitamin D level was 1.34 (95% CI 0.88-2.05; *P* = .18) for individuals with vitamin D level less than 20 ng/mL, 0.97 (95% CI, 0.62-1.52; *P* = .90) for individuals with a vitamin D level of 20 to less than 30 ng/mL, and 1.57 (95% CI, 1.06-2.33; *P* = .02) for individuals with a vitamin D level of 30 to less than 40 ng/mL, compared with those with a vitamin D level of 40 ng/mL or greater. In the model controlling for time since last vitamin D level measurement to estimate the association of vitamin D level 14 days before COVID-19 testing with test results, the IRR was 2.64 (95% CI, 1.24-5.66; *P* = .01) for individuals with vitamin D level less than 20 ng/mL, 1.69 (95% CI, 0.75-3.84; *P* = .21) for individuals with a vitamin D level of 20 to less than 30 ng/mL, and 2.55 (95% CI, 1.26-5.15; *P* = .009) for individuals with a vitamin D level of 30 to less than 40 ng/mL, compared with individuals with a vitamin D level of 40 ng/mL or greater. Time since most recent measurement of vitamin D level was significantly associated with increased risk of positive test results. However, for individuals with vitamin D level less than 40 ng/mL, this was offset by a decreased risk of positive test results with increased time since the vitamin D level was measured. In the models limited to individuals with a vitamin D level of 30 ng/mL or greater, the IRR was 2.49 (95% CI, 1.17-5.28; *P* = .02) for individuals with a vitamin D level of 40 ng/mL, and a 1-ng/mL increase in vitamin D level decreased the risk of positive test results by 5% (IRR, 0.95 [95% CI, 0.91-0.98]; *P* = .003). None of these analyses were statistically significant for White individuals, although 95% CIs were too wide to exclude the presence of the associations seen for Black individuals (eTable 1 in the Supplement). Notably, White individuals who decreased their vitamin D treatment between the time of vitamin D measurement and 14 days before COVID-19 testing were at increased risk of testing positive for COVID-19 (model 1: IRR, 2.44 [95% CI, 1.01-5.90]; *P* = .05; model 2: IRR, 2.50 [95% CI, 1.03-6.09]; *P* = .04).

The Figure depicts estimated COVID-19 positivity rates based on the multivariable models accounting for time since vitamin D testing stratified by most recent vitamin D level for the full population and separately for Black and White individuals. Estimated COVID-19 positivity rates were lowest for the highest vitamin D levels in Black individuals but not in White individuals. Estimated COVID-19 positivity rates in Black individuals were 9.72% (95% CI, 6.74%-13.41%) for those with a vitamin D level less than 20 ng/mL, 6.47% (95% CI, 3.33%-10.28%) for individuals with a vitamin D level of 20 to less than 30 ng/mL, 10.10% (95% CI, 6.00%-15.47%) for individuals with a vitamin D level of 30 to less than 40 ng/mL, and 3.82% (95% CI, 1.78%-6.68%) for individuals with a vitamin D level of 40 ng/mL or greater.

**Figure.**
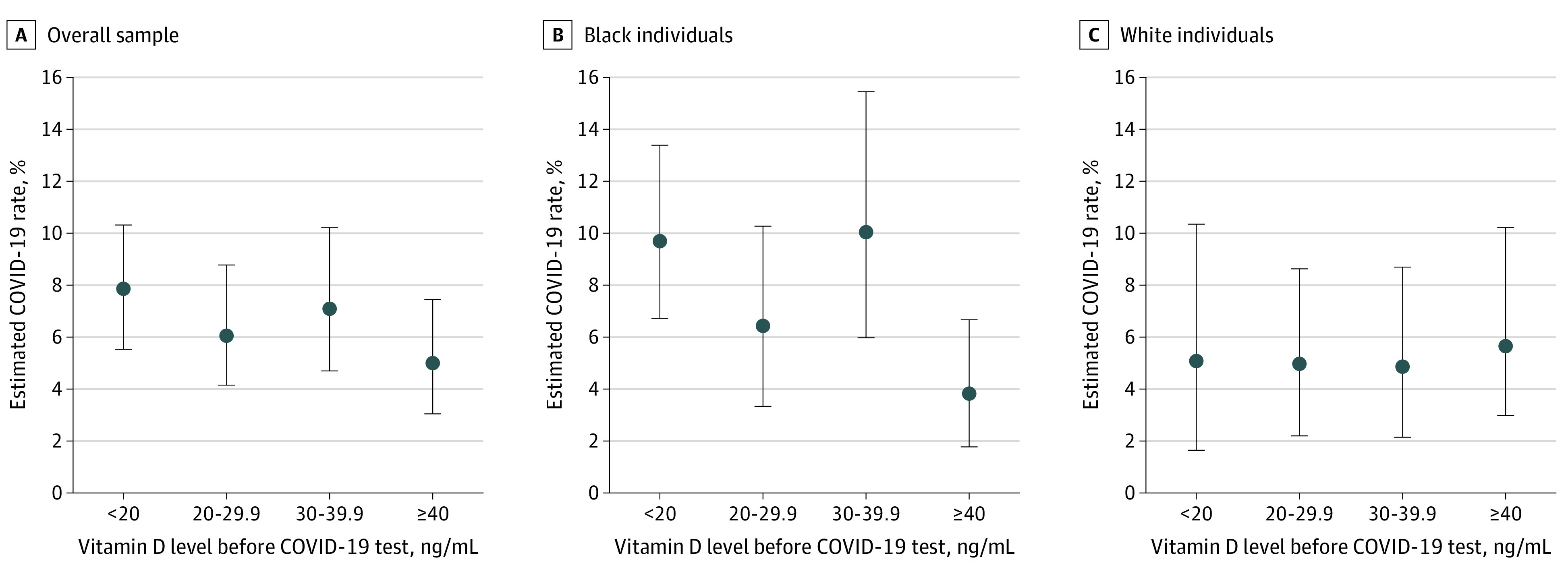
Estimated COVID-19 Rates by Most Recent Vitamin D Level To convert vitamin D to nanomoles per liter, multiply by 2.496.

Given the overall trend for a vitamin D level of 20 to less than 30 ng/mL to be less strongly associated with COVID-19 risk than a level of 30 to less than 40 ng/mL, we tested whether treatments were more likely to be increased if levels were lower. Indeed, for individuals reported as using vitamin D_3_ supplements on the date of their most recently measured vitamin D level, only 5 of 188 individuals (3%) with a vitamin D level of 30 to less than 40 ng/mL had a dose increase within 60 days of vitamin D measurement vs 15 of 189 individuals (8%) with a vitamin D level of 20 to less than 30 ng/mL (*P* = .04).

### Results of Sensitivity Analysis

eTable 2 in the Supplement reports rates of positive COVID-19 test results stratified by most recent vitamin D level and days since that level was measured. The sensitivity analysis reinforces our main finding that COVID-19 positivity rates were lowest for individuals whose vitamin D level was measured most recently and was 40 ng/mL or greater and highlights that positivity rates increased for individuals whose vitamin D level was measured most recently or 1 year prior and was found to be less than 20 ng/mL. Stratifying this analysis by race, this outcome was evident only among Black individuals. eTable 3 and eTable 4 in the Supplement report results for the full sample stratified by whether the person was using a vitamin D supplement 14 days before COVID-19 testing. Vitamin D levels were associated with positive test results only in individuals using supplements, and Hispanic ethnicity was associated with increased risk only in individuals not using supplements.

Table 4 reports rates of positive COVID-19 test results stratified by most recent vitamin D level and vitamin D_3_ dose 14 days before COVID-19 testing. COVID-19 positivity rates remained lowest for individuals with a vitamin D level of 40 ng/mL or greater. Among 1046 individuals with a vitamin D level of 40 or greater, 788 individuals (75%) did not use vitamin D supplements. Overall, only 920 of 4258 individuals (22%) used vitamin D_3_ supplements, 304 individuals (7%) used more than 1000 IU/d vitamin D_3_, and 131 individuals (3%) used more than 2000 IU/d. Among 131 individuals using 2001 IU/d vitamin D_3_ or more, 4 of 24 individuals (17%) with vitamin D level less than 20 ng/mL had test results positive for COVID-19, while 2 of 107 individuals (2%) with a vitamin D level of 20 ng/mL or higher had test results positive for COVID-19 (*P* = .01). Among persons with a vitamin D level of 20 or greater, 2 of 107 individuals (2%) using 2001 IU/d or more vitamin D_3_ had test results positive for COVID-19, compared with 197 of 3075 individuals (6%) using no vitamin D or lower doses having test results positive for COVID-19 (*P* = .06). Among 24 individuals with vitamin D level less than 20 ng/mL who reported using 2001 IU/d or more vitamin D_3_, 4 (17%) had test results positive for COVID-19; while among 1052 individuals who reported using 2000 IU/d or less vitamin D_3_, 96 (9%) had test results positive for COVID-19 (*P* = .27).

**Table 4.** COVID-19 Positivity Rate by Most Recent Vitamin D Level and by Treatment

Active treatment 14 d before first COVID-19 test order date	Individuals with positive COVID-19 test result/total tested, No. (%)				
	Total	Most recent vitamin D level from 14 to 365 d before first COVID-19 test order, ng/mL			
		<20	20-29	30-39	≥40
Total	299/4258 (7.0)	100/1076 (9.3)	70/1175 (6.0)	72/961 (7.5)	57/1046 (5.4)
None	239/3338 (7.2)	81/900 (9.0)	53/917 (5.8)	59/733 (8.0)	46/788 (5.8)
1-1000 IU/d D_3_ or multivitamin	45/616 (7.3)	13/116 (11.2)	13/171 (7.6)	12/167 (7.2)	7/162 (4.3)
1001-2000 IU/d D_3_	9/173 (5.2)	2/36 (5.6)	4/57 (7.0)	0/39	3/41 (7.3)
≥2001 IU/d D_3_	6/131 (4.6)	4/24 (16.7)	0/30	1/22 (4.5)	1/55 (1.8)

Abbreviation: COVID-19, coronavirus disease 2019.

Conversion factor: To convert vitamin D to nanomoles per liter, multiply by 2.496.

As only 239 individuals (5%) in total had a vitamin D level of 60 ng/mL or greater, we do not report multivariable analysis with this category. However, among 858 individuals with a vitamin D level of 40 to less than 60 ng/mL, 56 (7%) had test results positive for COVID-19, and among 239 individuals with a vitamin D level of 60 ng/mL or greater, 5 (2%) had test results positive for COVID-19 (*P* = .008). Of 11 individuals with a vitamin D level of 70 ng/mL or higher, none had positive COVID-19 test results, compared with 5 individuals (4%) with positive COVID-19 test results among 128 individuals with a vitamin D level of 60 to less than 70 ng/mL (*P* = .04).

## Discussion

This findings of this cohort study reinforce prior findings by ourselves and others^11,12,13^ that lower vitamin D levels (eg, <20 ng/mL) are associated with increased risk of having test results positive for COVID-19 and provides evidence that COVID-19 risk is also increased for Black individuals with a vitamin D level of 30 to less than 40 ng/mL compared with individuals with a vitamin D level of 40 ng/mL or greater. Risk of positive COVID-19 test results decreased significantly with increased vitamin D level of 30 ng/mL or greater when measured as a continuous variable. Our findings that controlling for time since last vitamin D level measurement increased the reduction in the odds of positive test results with increased vitamin D levels and that the risk of positive test results with lower vitamin D levels increased when those levels were more recent relative to the timing of COVID-19 testing support the idea that vitamin D level at the time of testing is most strongly associated with COVID-19 risk. Our finding that COVID-19 positivity rates were increased among individuals with a vitamin D level of less than 20 ng/mL measured 275 to 365 days before COVID-testing may reflect seasonal vitamin D variation in this group.

These findings add to the evolving literature demonstrating associations between vitamin D levels and COVID-19 risks and outcomes. As noted, a meta-analysis by Martineau et al^6^ reported vitamin D supplementation was associated with reducing other viral respiratory infections, even in people with vitamin D levels that were sufficient by current standards based largely on needs for bone health. All-cause mortality and other outcomes may be better with vitamin D levels of 40 to 60 ng/mL, at least in some racial groups.^3,26,27^ Our findings that vitamin D levels measured a longer period from COVID-19 testing were less associated with COVID-19 risk and that the association of vitamin D levels with risk were strongest in Black individuals may help explain why a 2020 study by Hastie et al^28^ using the UK Biobank did not find a statistically significant association between vitamin D levels 10 to 14 years before the COVID-19 testing and the risk of having positive test results.

Our finding that vitamin D dose increases were more likely when the level was 20 to less than 30 ng/mL compared with a level of 30 to less than 40 ng/mL may explain the otherwise surprising result that a level of 20 to less than 30 ng/mL was less strongly associated with increased COVID-19 risk than a level of 30 to less than 40 ng/mL. This possible explanation would be particularly important if treatment is not fully captured in the electronic health record, which is likely given the availability of vitamin D over the counter.

The significant association of vitamin D levels with COVID-19 risk in Black individuals that was not found in White individuals could reflect their higher COVID-19 risk, to which socioeconomic factors and structural inequities clearly contribute.^29^ Biological susceptibility to vitamin D deficiency may also be less frequent in White than Black individuals, since lighter skin increases vitamin D production in response to sunlight^30^ and vitamin D binding proteins may vary by race and affect vitamin D bioavailability.^31,32^ Many persons in our other race category, consisting larging of Asian or Mideast Indian individuals and individuals with multiple races, come from racial groups with higher incidence of vitamin D deficiency, but this group was too small to support multivariable analysis in our sample. Nevertheless, Hispanic ethnicity was associated with increased risk in some multivariable analyses (eTable 3 in the Supplement). We note also that our analysis for White individuals lacked statistical power to exclude large associations of vitamin D levels with COVID-19 risk.

That vitamin D levels were associated with COVID-19 positivity rates, especially in individuals using vitamin D supplements, supports the idea that supplementation might decrease COVID-19 risk by increasing vitamin D levels. Our finding of lower rates of positive COVID-19 test results in individuals with vitamin D levels of 20 ng/mL or greater using at least 2001 IU/d vitamin D_3_ supplements compared with individuals with levels of 20 ng/mL or greater using less than 2001 IU/d vitamin D_3_ supplements could reflect the effect of those higher doses on levels and therefore COVID-19 risk. The findings of increased risk for White individuals who decreased treatment after their vitamin D levels were tested also supports the potential effectiveness of vitamin D supplementation. It has also been hypothesized that free vitamin D levels may be more important to immune function than vitamin D levels and that free levels may be more affected by vitamin D intake or synthesis in some individuals since vitamin D may not be easily mobilized from vitamin D binding protein in some circumstances.^33^ This may explain our findings that vitamin D supplement use independent of vitamin D levels or potential proxies for unmeasured supplementation (eg, crossing into the 20 to <30 ng/mL insufficient range) are associated with decreased COVID-19 risk, and published findings that more frequent vitamin D dosing is more effective than intermittent dosing in improving immune function.^34^

These results increase the urgency to consider whether increased sun exposure or vitamin D supplementation could reduce COVID-19 risk. It is likely that less than 5% to 10% of US adults currently have vitamin D levels greater than 40 ng/mL.^13,35^ When increased sun exposure is impractical, achieving vitamin D levels of 40 ng/mL or greater typically requires greater supplementation than currently recommended for most individuals of 600-800 IU/d vitamin D_3_. The National Academy of Medicine’s highest recommended dose of 4000 IU/d vitamin D_3_ would substantially increase the fraction of people eventually reaching such levels, but more than 25% of individuals might not reach such levels,^36^ especially if they are obese or vitamin D deficient.^37^ Sustained use of 10 000 IU/d vitamin D_3_ would likely decrease the fraction of people with vitamin D levels less than 40 ng/mL to less than 5%. Such doses may increase the risk of hypercalcemia, but the risk of hypercalcemia with 10 000 IU/d vitamin D_3_ is low,^36,38^ can be mitigated by monitoring, and should be weighed against potential benefits. These arguments suggest that randomized clinical trials to understand if vitamin D can reduce COVID-19 risk should include doses likely to increase vitamin D to at least 40 ng/mL. Although limited by sample size that precludes multivariable analysis, our findings of lower COVID-19 positivity rates for individuals with vitamin D levels of 60 ng/mL or higher compared with lower levels suggest that randomized clinical trials are needed to study interventions to increase vitamin D levels to greater than 60 or even 70 ng/mL, which would increase the importance of considering safety concerns. Studies should also consider the role of vitamin D testing, loading doses, dose adjustments for individuals who are obese or overweight, risks for hypercalcemia, and strategies to monitor for and mitigate hypercalcemia, and that non-White populations, such as Black individuals, may have greater needs for supplementation, especially given their greater rates of vitamin D deficiency and disproportionate burden of COVID-19 morbidity and mortality. Cost and the potential for rapid scalability may also be important concerns, especially since vitamin D may cost less than $10 per year while screening may cost more than $50 and carry COVID-19 exposure risk for individuals not otherwise undergoing phlebotomy or unable to complete home testing. Since Medicare and Medicaid often do not cover the costs of screening for vitamin D deficiency, the increased rates of vitamin D deficiency in older adults and in racial minority groups in the United States, especially Black and Hispanic persons,^1^ and increased rates of economic disadvantage and COVID-19 in these groups^39^ suggest the need to reexamine these payment policies in the current context.

While the increasing availability of COVID-19 vaccines is likely to reduce the spread of COVID-19 and hence any potential benefits of vitamin D supplementation, the presence of new strains resistant to existing vaccines may increase the potential benefits of vitamin D supplementation, and vitamin D supplementation may be useful in populations not receiving the vaccine. Weakened host responses may also enhance the conditions for development of viral mutation,^40^ so supplementation might have benefits at the population level in reducing the risk of mutant strains.

### Limitations

This study has limitations. Despite basic scientific evidence of effects of vitamin D on innate and adaptive immunity, immunomodulation, and thrombotic regulation^3,41,42^ and the clinical trial evidence of effects of vitamin D supplementation on viral respiratory tract infections, the observed associations could be affected by omitted confounders so that they may not reflect causal effects of vitamin D deficiency on COVID-19 risk. However, the results are robust to including demographic characteristics and comorbidity indicators that have either physiological reasons for consideration or have been suggested to be associated with COVID-19 outcomes. Since our data are limited to the UCM electronic health record, patterns of vitamin D screening, treatment, or COVID-19 testing might have somehow selected for participants in a manner that induced an association between observed vitamin D status and testing positive for COVID-19. However, studies in many different clinical settings have now reported low vitamin D levels to be associated with increased COVID-19 risk.^6,7,8,10^ Our analyses are also limited by the possibility that vitamin D supplement use recorded in the electronic health record might not capture nonprescription vitamin D supplements and that individuals may not use supplements as reported in the electronic health record. Our analysis of the association of dosing with rates of COVID-19 positivity was also limited to individuals not using vitamin D supplements or vitamin D_3_ alone, excluding individuals using vitamin D_2_ or calcitriol, which are both frequently given in subgroups (eg, individuals with chronic kidney disease, hypoparathyroidism) deserving of separate analysis. Our study population from a northern city contained many Black individuals, older adults with chronic illness, and health care workers, all of which are risk factors for vitamin D deficiency^1,43,44,45,46,47^ and hence may not represent other populations. Related to this, larger sample sizes may be needed to establish if vitamin D levels affects COVID-19 risk in populations with lower risks of vitamin D deficiency or COVID-19, including White individuals and those in warmer climates where sun-related vitamin D production may be greater.

## Conclusions

The findings of this cohort study support a role of vitamin D levels in COVID-19 risk. Randomized clinical trials of interventions to raise vitamin D levels into ranges at or above levels currently considered sufficient are needed to determine if those interventions could reduce COVID-19 incidence, perhaps especially in Black and other populations known to be at increased risk of vitamin D deficiency. Because such levels exceed levels recommended for other reasons, individual and policy decisions about higher supplement dosing and vitamin D testing to achieve such levels should be even more carefully considered than dosing to avoid vitamin D deficiency as currently defined, which some have argued should be pursued given current evidence that vitamin D might reduce the risk of COVID-19.^48,49^

## Notes

Supplement. **eTable 1.** Multivariable Association of Vitamin D Levels and Treatment with Test Results Positive for COVID-19 Among Individuals Reporting White Race **eTable 2.** COVID-19 Test Positivity Rate by Most Recent Vitamin D Level and by Time Since Most Recent Measurement **eTable 3.** Multivariable Association of Vitamin D Levels and Treatment with Test Results Positive for COVID-19 Among Individuals With No Active Vitamin D Treatment Before COVID-19 Test **eTable 4.** Multivariable Association of Vitamin D Levels and Treatment with Test Results Positive for COVID-19 Among Individuals With Active Vitamin D Treatment Before COVID-19 Test **eFigure.** COVID-19 Test Positivity Rate by Race and by Most Recent Vitamin D Levels Before COVID-19 Tests **eAppendix.** Supplemental Methods
