# Supplementary material for: Association of Vitamin D Levels, Race/Ethnicity, and Clinical Characteristics With COVID-19 Test Results
Source: JAMA Netw Open. 2021 Mar 19;4(3):e214117. doi: 10.1001/jamanetworkopen.2021.4117 (PMC7980095; doi:10.1001/jamanetworkopen.2021.4117)
Supplement: Supplement. — eTable 1. Multivariable Association of Vitamin D Levels and Treatment with Test Results Positive for COVID-19 Among Individuals Reporting White Race eTable 2. COVID-19 Test Positivity Rate by Most Recent Vitamin D Level and by Time Since Most Recent Measurement eTable 3. Multivariable Association of Vitamin D Levels and Treatment with Test Results Positive for COVID-19 Among Individuals With No Active Vitamin D Treatment Before COVID-19 Test eTable 4. Multivariable Association of Vitamin D Levels and Treatment with Test Results Positive for COVID-19 Among Individuals With Active Vitamin D Treatment Before COVID-19 Test eFigure. COVID-19 Test Positivity Rate by Race and by Most Recent Vitamin D Levels Before COVID-19 Tests eAppendix. Supplemental Methods [file jamanetwopen-e214117-s001.pdf]

Supplemental Online Content

Meltzer DO, Best TJ, Zhang H, Vokes T, Arora VM, Solway J. Association of vitamin D levels, race/ethnicity, and other clinical characteristics with COVID-19 test results. *JAMA Netw Open*. 2021;4(3):e214117. doi:10.1001/jamanetworkopen.2021.4117

eTable 1. Multivariable Association of Vitamin D Levels and Treatment with Test Results Positive for COVID-19 Among Individuals Reporting White Race
eTable 2. COVID-19 Test Positivity Rate by Most Recent Vitamin D Level and by Time Since Most Recent Measurement
eTable 3. Multivariable Association of Vitamin D Levels and Treatment with Test Results Positive for COVID-19 Among Individuals With No Active Vitamin D Treatment Before COVID-19 Test
eTable 4. Multivariable Association of Vitamin D Levels and Treatment with Test Results Positive for COVID-19 Among Individuals With Active Vitamin D Treatment Before COVID-19 Test
eFigure. COVID-19 Test Positivity Rate by Race and by Most Recent Vitamin D Levels Before COVID-19 Tests
eAppendix. Supplemental Methods

This supplemental material has been provided by the authors to give readers additional information about their work.

© 2021 Meltzer DO et al. *JAMA Network Open.*

eTable 1. Multivariable Association of Vitamin D Levels and Treatment with Test Results Positive for COVID-19 Among Individuals Reporting White Race

	Model 1 (M1) and M2		M1		M2: M1 plus interaction between most recent calcifediol level and months since that level[†]		M3 and M4		M3: M2 restricted to persons whose most recent calcifediol level ≥30 ng/ml		M4: M3, except level is now included as a continuous variable (i.e., not categorized)	
	N	%	Incidence Rate Ratio (IRR) (95% CI)	p	IRR (95% CI)	p	N	%	IRR (95% CI)	p	IRR (95% CI)	p
Number of persons	1999	100					1108	100				
Gender (Male is reference)	680	34					354	32				
Female	1319	66	0.75 (0.49, 1.15)	.19	0.74 (0.48, 1.15)	.18	754	68	0.97 (0.52, 1.80)	.91	0.99 (0.53, 1.84)	.97
Ethnicity (Not Hispanic is reference)	1818	91					1034	93				
Hispanic	181	9	1.19 (0.67, 2.13)	.56	1.20 (0.67, 2.15)	.54	74	7	1.23 (0.49, 3.05)	.66	1.22 (0.49, 3.06)	.67
UChicago employee status (Not employee is reference)	1727	86					971	88				
UChicago Employee	272	14	1.52 (0.93, 2.49)	.09	1.55 (0.94, 2.54)	.08	137	12	2.13 (1.17, 3.87)	.01	2.08 (1.13, 3.84)	.02
Most recent calcifediol level before COVID-19 test (ng/ml) (mean, std. dev.)	33.9	15.3					44.0	12.7			0.98 (0.94, 1.01)	.18
≥40 (reference)	602	30					602	54				
30–39	506	25	1.13 (0.69, 1.84)	.63	0.86 (0.38, 1.96)	.72	506	46	0.87 (0.37, 2.01)	.74		
20–29	576	29	0.97 (0.58, 1.61)	.90	0.88 (0.37, 2.06)	.76						
<20	315	16	1.14 (0.63, 2.07)	.66	0.89 (0.34, 2.35)	.82						
Months since most recent calcifediol level (mean, std. dev.)[†]	5.5	3.4			0.96 (0.87, 1.07)	.51	5.3	3.5	0.96 (0.86, 1.06)	.42	0.94 (0.70, 1.27)	.69
Interactions between (Most recent calcifediol level before COVID-19 test) and (Months since most recent calcifediol level)											1.00 (0.99, 1.01)	.73
(30–39) * (Months since most recent calcifediol level)					1.06 (0.92, 1.23)	.41			1.06 (0.92, 1.23)	.44		
(20–29) * (Months since most recent calcifediol level)					1.03 (0.88, 1.19)	.74						
(<20) * (Months since most recent calcifediol level)					1.05 (0.90, 1.24)	.52						
Vitamin D dose change after most recent calcifediol level?*												
Increased	155	8	1.08 (0.51, 2.27)	.84	1.08 (0.52, 2.28)	.83	64	6	0.63 (0.16, 2.39)	.49	0.65 (0.17, 2.55)	.54
Decreased	60	3	2.44 (1.01, 5.90)	.05	2.50 (1.03, 6.09)	.04	26	2	omitted[§]		omitted[§]	
Age (mean, std. dev.)	51.7	19.1					55.2	18.8				
Demeaned age			0.98 (0.95, 1.02)	.35	0.98 (0.95, 1.02)	.35			0.98 (0.93, 1.02)	.29	0.98 (0.93, 1.02)	.32
Time period of COVID-19 test (December: reference)	156	8					93	8				
March	101	5	0.90 (0.35, 2.29)	.83	0.91 (0.36, 2.33)	.85	51	5	0.55 (0.07, 4.56)	.58	0.51 (0.06, 4.21)	.53
April	125	6	0.47 (0.16, 1.37)	.16	0.48 (0.17, 1.38)	.17	53	5	0.48 (0.05, 4.13)	.50	0.45 (0.05, 3.96)	.48
May	206	10	0.46 (0.18, 1.16)	.10	0.47 (0.19, 1.17)	.10	107	10	1.90 (0.63, 5.75)	.25	1.78 (0.60, 5.34)	.30
June	265	13	0.19 (0.06, 0.62)	.006	0.19 (0.06, 0.63)	.006	147	13	0.56 (0.11, 3.00)	.50	0.54 (0.10, 2.87)	.47
July	287	14	0.19 (0.07, 0.57)	.003	0.20 (0.07, 0.57)	.003	144	13	0.78 (0.23, 2.71)	.70	0.79 (0.23, 2.72)	.71
August	219	11	0.18 (0.06, 0.55)	.003	0.18 (0.06, 0.55)	.003	121	11	omitted[§]		omitted[§]	
September	171	9	0.05 (0.01, 0.29)	<.001	0.05 (0.01, 0.29)	<.001	95	9	0.15 (0.02, 1.12)	.06	0.15 (0.02, 1.08)	.06
October	255	13	1.06 (0.52, 2.17)	.88	1.07 (0.52, 2.19)	.85	162	15	2.60 (1.03, 6.55)	.04	2.52 (0.99, 6.41)	.05
November	214	11	1.84 (0.93, 3.65)	.08	1.88 (0.94, 3.75)	.07	135	12	3.29 (1.30, 8.33)	.01	3.13 (1.24, 7.93)	.02
Interactions between (Time period of COVID-19 test) and (Demeaned age)												

© 2021 Meltzer DO et al. *JAMA Network Open.*

(December * (Demeaned age): reference)												
March * (Demeaned age)			1.04 (0.99, 1.09)	.10	1.04 (0.99, 1.09)	.10			1.09 (1.04, 1.15)	<.001	1.09 (1.04, 1.14)	<.001
April * (Demeaned age)			1.01 (0.94, 1.09)	.74	1.01 (0.94, 1.09)	.72			1.00 (0.96, 1.05)	.88	1.00 (0.96, 1.05)	.85
May * (Demeaned age)			1.01 (0.97, 1.06)	.53	1.02 (0.97, 1.06)	.52			1.02 (0.96, 1.08)	.58	1.02 (0.96, 1.08)	.60
June * (Demeaned age)			0.97 (0.92, 1.03)	.29	0.97 (0.92, 1.03)	.30			0.96 (0.90, 1.04)	.31	0.96 (0.90, 1.03)	.29
July * (Demeaned age)			0.99 (0.95, 1.04)	.71	0.99 (0.95, 1.04)	.73			1.00 (0.95, 1.06)	.89	1.00 (0.95, 1.05)	.94
August * (Demeaned age)			0.96 (0.92, 1.01)	.10	0.96 (0.92, 1.01)	.11						
September * (Demeaned age)			0.93 (0.89, 0.99)	.01	0.93 (0.89, 0.99)	.01			0.96 (0.91, 1.01)	.10	0.96 (0.91, 1.01)	.09
October * (Demeaned age)			1.03 (0.99, 1.07)	.21	1.03 (0.98, 1.07)	.21			1.03 (0.98, 1.08)	.25	1.03 (0.98, 1.08)	.26
November * (Demeaned age)			1.02 (0.98, 1.06)	.37	1.02 (0.98, 1.06)	.35			1.04 (0.99, 1.09)	.14	1.04 (0.99, 1.09)	.14
Comorbidity indicators												
Hypertension	685	34	1.20 (0.72, 1.98)	.49	1.20 (0.73, 1.98)	.46	404	36	1.35 (0.73, 2.50)	.34	1.34 (0.72, 2.50)	.36
Comorbidities with immunosuppression	491	25	0.88 (0.53, 1.48)	.64	0.88 (0.53, 1.46)	.62	281	25	0.84 (0.44, 1.58)	.59	0.87 (0.46, 1.65)	.68
Diabetes	322	16	1.55 (0.94, 2.55)	.08	1.54 (0.93, 2.55)	.09	177	16	1.29 (0.66, 2.53)	.46	1.32 (0.67, 2.61)	.42
Renal failure	207	10	0.79 (0.35, 1.77)	.57	0.79 (0.35, 1.77)	.57	114	10	1.10 (0.41, 2.94)	.85	1.15 (0.41, 3.18)	.79
Chronic pulmonary disease	267	13	0.59 (0.28, 1.28)	.18	0.60 (0.28, 1.28)	.19	153	14	0.40 (0.12, 1.29)	.13	0.39 (0.12, 1.25)	.11
Depression	298	15	0.93 (0.55, 1.57)	.79	0.93 (0.55, 1.57)	.78	165	15	0.90 (0.42, 1.95)	.79	0.88 (0.41, 1.90)	.74
Liver disease	250	13	1.07 (0.58, 1.98)	.82	1.08 (0.58, 2.02)	.80	139	13	0.77 (0.31, 1.92)	.57	0.77 (0.30, 1.95)	.58
Psychoses	101	5	0.86 (0.35, 2.13)	.75	0.87 (0.35, 2.12)	.75	61	6	0.38 (0.06, 2.37)	.30	0.37 (0.06, 2.37)	.29
Pulmonary circulation disorders	75	4	1.32 (0.56, 3.14)	.53	1.29 (0.54, 3.07)	.56	45	4	1.03 (0.24, 4.48)	.97	1.02 (0.23, 4.50)	.98
Dementia	9	0	2.52 (0.32, 20.00)	.38	2.57 (0.32, 20.40)	.37	6	1	omitted[ξ]		omitted[ξ]	
BMI (kg/m^2) (mean, std. dev.)	28.1	7.1	1.02 (0.99, 1.05)	.18	1.02 (0.99, 1.05)	.18	27.6	6.7	1.02 (0.98, 1.06)	.34	1.02 (0.98, 1.05)	.39
Goodness-of-link test of squared predicted value				.33		.43				.60		.15
Hosmer-Lemeshow goodness-of-fit decile test				.86		.77				.85		.63

* Vitamin D dose was rank-ordered as follows: Calcitriol > 4001+ IU D3 > 2001-4000 IU D3 > 1001-2000 IU D3 > D2 > 1-1000 IU D3/Multivitamin > No Vitamin D.

† Months since most recent calcifediol level was defined as the number of days between their most recent calcifediol level and 14 days before their first COVID-19 test order, divided by (365/12).

ξ Stata did not produce a reliable coefficient estimate for this covariate because 0 of the persons in the subgroup tested positive for COVID-19, so we omitted the covariate as indicated.

© 2021 Meltzer DO et al. *JAMA Network Open.*

eTable 2. COVID-19 Test Positivity Rate by Most Recent Vitamin D Level and by Time Since Most Recent Measurement

Overall sample	Most recent calcifediol level from 14 to 365 days before first COVID-19 test order				
Days since most recent calcifediol level	Total	<20 ng/mL	20 – 29 ng/mL	30 – 39 ng/mL	≥40 ng/mL
14 - 91	106/1419 (7.5)	40/348 (11.5)	24/367 (6.5)	24/334 (7.2)	18/370 (4.9)
92 - 182	85/1219 (7.0)	27/361 (7.5)	23/331 (6.9)	21/239 (8.8)	14/288 (4.9)
183 - 274	65/1058 (6.1)	19/292 (6.5)	12/304 (3.9)	18/235 (7.7)	16/227 (7.0)
275 - 365	77/942 (8.2)	32/250 (12.8)	17/265 (6.4)	15/215 (7.0)	13/212 (6.1)
Total	333/4638 (7.2)	118/1251 (9.4)	76/1267 (6.0)	78/1023 (7.6)	61/1097 (5.6)
Black	Most recent calcifediol level from 14 to 365 days before first COVID-19 test order				
Days since most recent calcifediol level	Total	<20 ng/mL	20 – 29 ng/mL	30 – 39 ng/mL	≥40 ng/mL
14 - 91	65/681(9.5)	33/243 (13.6)	15/165 (9.1)	13/138 (9.4)	4 /135 (3.0)
92 - 182	55/616 (8.9)	23/240 (9.6)	10/159 (6.3)	15/108 (13.9)	7/109 (6.4)
183 - 274	39/539 (7.2)	11/183 (6.0)	8/147 (5.4)	9/112 (8.0)	11/97 (11.3)
275 - 365	52/452 (11.5)	27/163 (16.6)	11/115 (9.6)	7/83 (8.4)	7/91 (7.7)
Total	211/2288 (9.2)	94/829 (11.3)	44/586 (7.5)	44/441 (10.0)	29/432 (6.7)
White	Most recent calcifediol level from 14 to 365 days before first COVID-19 test order				
Days since most recent calcifediol level	Total	<20 ng/mL	20 – 29 ng/mL	30 – 39 ng/mL	≥40 ng/mL
14 - 91	33/622 (5.3)	5/74 (6.8)	6/165 (3.6)	10/170 (5.9)	12/213 (5.6)
92 - 182	24/506 (4.7)	2/93 (2.2)	11/146 (7.5)	4/102 (3.9)	7/165 (4.2)
183 - 274	24/460 (5.2)	6/85 (7.1)	4/141 (2.8)	9/116 (7.8)	5/118 (4.2)
275 - 365	21/411 (5.1)	4/63 (6.3)	6/124 (4.8)	6/118 (5.1)	5/106 (4.7)
Total	102/1999 (5.1)	17/315 (5.4)	27/576 (4.7)	29/506 (5.7)	29/602 (4.8)

© 2021 Meltzer DO et al. *JAMA Network Open.*

eTable 3. Multivariable Association of Vitamin D Levels and Treatment with Test Results Positive for COVID-19 Among Individuals With No Active Vitamin D Treatment Before COVID-19 Test

	Model 1 (M1) and M2		M1		M2: M1 plus interaction between most recent calcifediol level and months since that level[†]		M3 and M4		M3: M2 restricted to persons whose most recent calcifediol level ≥30 ng/ml		M4: M3, except level is now included as a continuous variable (i.e., not categorized)	
	N	%	Incidence Rate Ratio (IRR) (95% CI)	p	IRR (95% CI)	p	N	%	IRR (95% CI)	p	IRR (95% CI)	p
Number of persons	3338	100					1521	100				
Gender (Male is reference)	999	30					442	29				
Female	2339	70	0.70 (0.53, 0.91)	.008	0.70 (0.53, 0.91)	.009	1079	71	0.71 (0.48, 1.06)	.10	0.74 (0.50, 1.10)	.13
Race (White is reference)	1493	45					817	54				
Black	1597	48	1.62 (1.20, 2.20)	.002	1.62 (1.19, 2.20)	.002	608	40	1.31 (0.84, 2.04)	.23	1.32 (0.85, 2.05)	.21
Other	248	7	0.87 (0.52, 1.47)	.61	0.87 (0.52, 1.46)	.59	96	6	0.90 (0.37, 2.17)	.81	0.87 (0.36, 2.09)	.75
Ethnicity (Not Hispanic is reference)	3129	94					1443	95				
Hispanic	209	6	1.89 (1.18, 3.04)	.008	1.90 (1.18, 3.04)	.008	78	5	1.70 (0.80, 3.63)	.17	1.72 (0.80, 3.68)	.16
UChicago employee status (Not employee is reference)	2905	87					1356	89				
UChicago Employee	433	13	1.35 (0.96, 1.90)	.09	1.35 (0.96, 1.91)	.09	165	11	1.43 (0.85, 2.39)	.18	1.42 (0.85, 2.37)	.19
Most recent calcifediol level before COVID-19 test (ng/ml) (mean, std. dev.)	30.3	15.7					43.6	13.0			0.98 (0.95, 1.01)	.11
≥40 (reference)	788	24					788	52				
30–39	733	22	1.37 (0.95, 1.98)	.09	1.11 (0.59, 2.10)	.75	733	48	1.05 (0.56, 1.98)	.88		
20–29	917	27	0.91 (0.62, 1.34)	.63	1.02 (0.54, 1.93)	.96						
<20	900	27	1.39 (0.98, 1.99)	.07	1.20 (0.66, 2.19)	.54						
Months since most recent calcifediol level (mean, std. dev.)[†]	5.5	3.4			0.99 (0.92, 1.07)	.89	5.4	3.5	1.01 (0.94, 1.09)	.74	1.02 (0.84, 1.23)	.87
Interactions between (Most recent calcifediol level before COVID-19 test) and (Months since most recent calcifediol level)											1.00 (1.00, 1.01)	.85
(30–39) * (Months since most recent calcifediol level)					1.04 (0.94, 1.15)	.43			1.04 (0.94, 1.15)	.49		
(20–29) * (Months since most recent calcifediol level)					0.98 (0.88, 1.09)	.68						
(<20) * (Months since most recent calcifediol level)					1.03 (0.94, 1.13)	.55						
Vitamin D dose change after most recent calcifediol level?* (No change is reference)	3968	86					1451	95				
Decreased	193	4	1.18 (0.68, 2.04)	.55	1.19 (0.68, 2.07)	.54	70	5	0.29 (0.07, 1.17)	.08	0.32 (0.08, 1.29)	.11
Age (mean, std. dev.)	52.7	19.5					56.7	19.8				
Demeaned age			0.99 (0.97, 1.00)	.12	0.99 (0.97, 1.00)	.12			0.96 (0.94, 0.99)	.01	0.97 (0.94, 0.99)	.01
Time period of COVID-19 test (December: reference)	248	7					119	8				
March	232	7	1.60 (0.91, 2.80)	.10	1.61 (0.92, 2.82)	.10	81	5	1.38 (0.45, 4.26)	.57	1.34 (0.43, 4.19)	.62
April	278	8	1.52 (0.88, 2.60)	.13	1.52 (0.88, 2.60)	.13	108	7	2.19 (0.89, 5.38)	.09	2.17 (0.88, 5.34)	.09
May	363	11	0.86 (0.48, 1.52)	.59	0.86 (0.48, 1.53)	.61	156	10	1.45 (0.58, 3.62)	.43	1.42 (0.57, 3.56)	.45
June	436	13	0.22 (0.10, 0.50)	<.001	0.22 (0.10, 0.50)	<.001	199	13	0.27 (0.07, 1.01)	.05	0.28 (0.07, 1.03)	.06
July	439	13	0.18 (0.07, 0.44)	<.001	0.18 (0.07, 0.44)	<.001	184	12	0.27 (0.07, 1.06)	.06	0.28 (0.07, 1.09)	.07
August	348	10	0.25 (0.10, 0.63)	.003	0.25 (0.10, 0.63)	.003	178	12	0.09 (0.01, 1.24)	.07	0.09 (0.01, 1.23)	.07
September	261	8	0.15 (0.04, 0.49)	.002	0.14 (0.04, 0.48)	.002	125	8	0.17 (0.04, 0.82)	.03	0.17 (0.04, 0.83)	.03

© 2021 Meltzer DO et al. *JAMA Network Open.*

October	369	11	1.15 (0.67, 1.99)	.62	1.15 (0.67, 1.99)	.62	192	13	1.61 (0.67, 3.86)	.28	1.61 (0.67, 3.88)	.29
November	364	11	2.22 (1.36, 3.63)	.001	2.22 (1.36, 3.64)	.001	179	12	2.36 (1.03, 5.40)	.04	2.36 (1.02, 5.44)	.04
Interactions between (Time period of COVID-19 test) and (Demeaned age) (December * (Demeaned age): reference)												
March * (Demeaned age)			1.04 (1.02, 1.07)	<.001	1.04 (1.02, 1.07)	<.001			1.12 (1.06, 1.17)	<.001	1.12 (1.06, 1.17)	<.001
April * (Demeaned age)			1.02 (1.00, 1.05)	.05	1.02 (1.00, 1.05)	.05			1.05 (1.02, 1.09)	.006	1.05 (1.01, 1.09)	.007
May * (Demeaned age)			1.01 (0.99, 1.04)	.30	1.01 (0.99, 1.04)	.27			1.02 (0.99, 1.06)	.19	1.02 (0.99, 1.06)	.20
June * (Demeaned age)			0.97 (0.94, 1.00)	.06	0.97 (0.94, 1.00)	.06			0.99 (0.95, 1.03)	.51	0.98 (0.94, 1.03)	.47
July * (Demeaned age)			0.98 (0.95, 1.02)	.37	0.98 (0.95, 1.02)	.38			1.02 (0.98, 1.06)	.32	1.02 (0.98, 1.06)	.32
August * (Demeaned age)			0.99 (0.94, 1.03)	.57	0.99 (0.94, 1.03)	.59			0.99 (0.91, 1.07)	.73	0.99 (0.91, 1.07)	.73
September * (Demeaned age)			1.02 (0.96, 1.08)	.60	1.02 (0.96, 1.08)	.59			0.99 (0.95, 1.02)	.38	0.98 (0.95, 1.02)	.33
October * (Demeaned age)			1.01 (0.99, 1.04)	.25	1.01 (0.99, 1.04)	.26			1.04 (1.00, 1.07)	.03	1.04 (1.00, 1.07)	.03
November * (Demeaned age)			1.02 (1.00, 1.04)	.09	1.02 (1.00, 1.04)	.09			1.05 (1.02, 1.08)	.003	1.05 (1.02, 1.08)	.002
Comorbidity indicators												
Hypertension	1638	49	1.12 (0.80, 1.57)	.52	1.12 (0.80, 1.57)	.52	787	52	1.30 (0.78, 2.18)	.31	1.29 (0.77, 2.16)	.33
Comorbidities with immunosuppression	836	25	0.78 (0.57, 1.06)	.11	0.77 (0.56, 1.05)	.10	408	27	0.51 (0.31, 0.83)	.007	0.52 (0.32, 0.86)	.01
Diabetes	786	24	1.10 (0.81, 1.49)	.54	1.09 (0.80, 1.48)	.57	374	25	0.91 (0.56, 1.47)	.69	0.91 (0.56, 1.47)	.70
Renal failure	647	19	1.07 (0.76, 1.49)	.71	1.07 (0.76, 1.49)	.70	318	21	1.06 (0.66, 1.71)	.80	1.08 (0.67, 1.75)	.74
Chronic pulmonary disease	613	18	0.98 (0.71, 1.36)	.92	0.97 (0.70, 1.35)	.88	270	18	1.13 (0.71, 1.78)	.61	1.16 (0.73, 1.83)	.53
Depression	535	16	1.11 (0.80, 1.54)	.52	1.10 (0.79, 1.53)	.56	221	15	1.10 (0.67, 1.79)	.71	1.10 (0.68, 1.78)	.71
Liver disease	397	12	0.90 (0.59, 1.36)	.60	0.91 (0.60, 1.38)	.66	182	12	0.94 (0.51, 1.73)	.83	0.93 (0.51, 1.71)	.81
Psychoses	205	6	0.72 (0.41, 1.25)	.25	0.72 (0.41, 1.26)	.24	94	6	0.84 (0.37, 1.89)	.67	0.81 (0.36, 1.84)	.62
Pulmonary circulation disorders	145	4	0.61 (0.30, 1.25)	.18	0.62 (0.30, 1.26)	.19	57	4	0.27 (0.04, 1.83)	.18	0.26 (0.04, 1.85)	.18
Dementia	69	2	1.41 (0.79, 2.51)	.24	1.40 (0.79, 2.50)	.25	36	2	1.50 (0.73, 3.08)	.27	1.48 (0.71, 3.10)	.29
BMI (kg/m^2) (mean, std. dev.)	29.4	8.1	1.00 (0.99, 1.02)	.74	1.00 (0.99, 1.02)	.75	28.3	7.2	1.01 (0.98, 1.03)	.65	1.00 (0.98, 1.03)	.79
Goodness-of-link test of squared predicted value				.80		.87				.94		.68
Hosmer-Lemeshow goodness-of-fit decile test				.30		.27				.31		.65

* Vitamin D dose was rank-ordered as follows: Calcitriol > 4001+ IU D3 > 2001-4000 IU D3 > 1001-2000 IU D3 > D2 > 1-1000 IU D3/Multivitamin > No Vitamin D.

† Months since most recent calcifediol level was defined as the number of days between their most recent calcifediol level and 14 days before their first COVID-19 test order, divided by (365/12).

© 2021 Meltzer DO et al. *JAMA Network Open.*

eTable 4. Multivariable Association of Vitamin D Levels and Treatment with Test Results Positive for COVID-19 Among Individuals With Active Vitamin D Treatment Before COVID-19 Test

	Model 1 (M1) and M2		M1		M2: M1 plus interaction between most recent calcifediol level and months since that level[†]		M3 and M4		M3: M2 restricted to persons whose most recent calcifediol level ≥30 ng/ml		M4: M3, except level is now included as a continuous variable (i.e., not categorized)	
	N	%	Incidence Rate Ratio (IRR) (95% CI)	p	IRR (95% CI)	p	N	%	IRR (95% CI)	p	IRR (95% CI)	p
Number of persons	1300	100					599	100				
Gender (Male is reference)	434	33					184	31				
Female	866	67	0.64 (0.42, 0.98)	.04	0.63 (0.41, 0.96)	.03	415	69	1.68 (0.65, 4.36)	.28	1.72 (0.69, 4.31)	.25
Race (White is reference)	506	39					291	49				
Black			1.23 (0.75, 2.02)	.41	1.21 (0.74, 1.98)	.44	265	44	1.34 (0.59, 3.04)	.49	1.36 (0.60, 3.07)	.46
Other	794	61	0.74 (0.25, 2.16)	.58	0.70 (0.24, 2.01)	.51	43	7	0.85 (0.17, 4.24)	.84	0.81 (0.18, 3.72)	.79
Ethnicity (Not Hispanic is reference)	1202	92					560	93				
Hispanic	98	8	0.50 (0.16, 1.62)	.25	0.50 (0.15, 1.59)	.24	39	7	0.84 (0.11, 6.56)	.87	0.88 (0.10, 7.62)	.91
UChicago employee status (Not employee is reference)	1176	90					538	90				
UChicago Employee	124	10	1.25 (0.60, 2.59)	.55	1.26 (0.60, 2.64)	.55	61	10	3.33 (1.31, 8.45)	.01	3.67 (1.43, 9.40)	.007
Most recent calcifediol level before COVID-19 test (ng/ml) (mean, std. dev.)	30.4	16.2					44.1	12.9			0.92 (0.87, 0.97)	.005
≥40 (reference)	309	24					309	52				
30–39	290	22	1.16 (0.61, 2.20)	.66	4.38 (1.36, 14.14)	.01	290	48	3.91 (1.20, 12.71)	.02		
20–29	350	27	1.12 (0.60, 2.09)	.72	2.55 (0.72, 9.02)	.15						
<20	351	27	1.59 (0.84, 3.01)	.16	3.67 (1.20, 11.24)	.02						
Months since most recent calcifediol level (mean, std. dev.)[†]	5.4	3.3			1.19 (1.04, 1.36)	.01	5.2	3.4	1.13 (0.98, 1.30)	.10	0.63 (0.45, 0.88)	.007
Interactions between (Most recent calcifediol level before COVID-19 test) and (Months since most recent calcifediol level)											1.01 (1.00, 1.02)	.008
(30–39) * (Months since most recent calcifediol level)					0.76 (0.64, 0.92)	.003			0.78 (0.65, 0.95)	.01		
(20–29) * (Months since most recent calcifediol level)					0.86 (0.72, 1.03)	.10						
(<20) * (Months since most recent calcifediol level)					0.86 (0.74, 1.00)	.05						
Vitamin D dose change after most recent calcifediol level?*												
Increased	477	37	0.88 (0.58, 1.34)	.56	0.86 (0.57, 1.32)	.50	133	22	0.95 (0.44, 2.07)	.90	1.03 (0.47, 2.23)	.95
Decreased	20	2	1.61 (0.44, 5.90)	.47	1.70 (0.46, 6.32)	.43	8	1	omitted[§]		omitted[§]	
Age (mean, std. dev.)	53.1	19.4					57.0	18.8				
Demeaned age			0.98 (0.95, 1.02)	.28	0.98 (0.95, 1.02)	.29			0.98 (0.90, 1.06)	.56	0.98 (0.90, 1.06)	.58
Time period of COVID-19 test (December: reference)	83	6					49	8				
March	91	7	0.85 (0.35, 2.08)	.72	0.81 (0.33, 1.99)	.64	29	5	1.14 (0.22, 5.88)	.88	0.92 (0.17, 4.89)	.92
April	153	12	1.27 (0.60, 2.70)	.53	1.18 (0.55, 2.50)	.67	52	9	3.55 (0.91, 13.88)	.07	3.59 (0.93, 13.85)	.06
May	182	14	0.35 (0.14, 0.89)	.03	0.32 (0.13, 0.80)	.01	69	12	0.34 (0.06, 1.89)	.22	0.30 (0.05, 1.71)	.17
June	188	14	0.16 (0.05, 0.52)	.002	0.15 (0.05, 0.49)	.002	91	15	0.28 (0.03, 2.55)	.26	0.27 (0.03, 2.38)	.24
July	168	13	0.13 (0.04, 0.49)	.002	0.12 (0.03, 0.45)	.002	82	14	0.20 (0.02, 2.54)	.22	0.22 (0.02, 2.07)	.19
August	105	8	0.19 (0.04, 0.86)	.03	0.17 (0.04, 0.80)	.02	48	8	0.52 (0.06, 4.56)	.55	0.47 (0.06, 3.55)	.47
September	101	8	0.09 (0.02, 0.48)	.005	0.08 (0.01, 0.42)	.003	57	10	omitted[§]		omitted[§]	

© 2021 Meltzer DO et al. *JAMA Network Open.*

October	114	9	0.59 (0.23, 1.54)	.28	0.50 (0.19, 1.33)	.17	60	10	1.14 (0.30, 4.32)	.84	1.05 (0.29, 3.87)	.94
November	115	9	1.04 (0.47, 2.31)	.92	0.94 (0.42, 2.08)	.87	62	10	1.84 (0.50, 6.70)	.36	1.70 (0.47, 6.17)	.42
Interactions between (Time period of COVID-19 test) and (Demeaned age) (December * (Demeaned age): reference)												
March * (Demeaned age)			1.05 (1.01, 1.10)	.02	1.06 (1.01, 1.10)	.02			1.13 (1.04, 1.23)	.006	1.12 (1.03, 1.22)	.01
April * (Demeaned age)			1.03 (1.00, 1.07)	.08	1.03 (1.00, 1.07)	.09			1.05 (0.96, 1.16)	.26	1.05 (0.96, 1.16)	.28
May * (Demeaned age)			1.02 (0.97, 1.07)	.46	1.02 (0.97, 1.07)	.46			1.12 (1.02, 1.23)	.02	1.12 (1.01, 1.23)	.03
June * (Demeaned age)			1.01 (0.97, 1.05)	.76	1.00 (0.97, 1.04)	.83			1.00 (0.92, 1.08)	.91	1.00 (0.91, 1.09)	.95
July * (Demeaned age)			0.97 (0.92, 1.02)	.27	0.97 (0.92, 1.02)	.24			0.93 (0.85, 1.03)	.17	0.94 (0.86, 1.03)	.19
August * (Demeaned age)			0.98 (0.92, 1.04)	.44	0.97 (0.91, 1.04)	.42			1.02 (0.94, 1.11)	.68	1.02 (0.93, 1.11)	.68
September * (Demeaned age)			0.95 (0.90, 1.00)	.07	0.95 (0.90, 1.00)	.07			omitted[ξ]		omitted[ξ]	
October * (Demeaned age)			1.01 (0.95, 1.07)	.75	1.01 (0.95, 1.07)	.77			1.04 (0.94, 1.15)	.42	1.04 (0.94, 1.15)	.43
November * (Demeaned age)			1.01 (0.97, 1.06)	.55	1.01 (0.97, 1.06)	.63			1.03 (0.93, 1.14)	.53	1.04 (0.94, 1.15)	.46
Comorbidity indicators												
Hypertension	729	56	1.26 (0.80, 1.97)	.32	1.23 (0.78, 1.96)	.38	337	56	1.78 (0.84, 3.80)	.13	1.82 (0.86, 3.87)	.12
Comorbidities with immunosuppression	333	26	0.71 (0.42, 1.19)	.20	0.69 (0.41, 1.15)	.15	152	25	0.80 (0.36, 1.74)	.57	0.85 (0.39, 1.86)	.69
Diabetes	370	28	1.22 (0.79, 1.88)	.37	1.22 (0.79, 1.87)	.37	167	28	1.17 (0.57, 2.40)	.66	1.19 (0.57, 2.50)	.64
Renal failure	291	22	0.88 (0.55, 1.40)	.58	0.90 (0.57, 1.44)	.66	133	22	0.69 (0.27, 1.78)	.45	0.71 (0.27, 1.88)	.49
Chronic pulmonary disease	309	24	0.62 (0.38, 1.00)	.05	0.62 (0.38, 1.00)	.05	141	24	0.36 (0.12, 1.08)	.07	0.40 (0.14, 1.13)	.08
Depression	261	20	1.55 (1.00, 2.39)	.05	1.46 (0.94, 2.27)	.09	105	18	2.23 (0.91, 5.50)	.08	2.40 (1.01, 5.70)	.05
Liver disease	217	17	1.05 (0.60, 1.82)	.86	1.03 (0.59, 1.78)	.92	110	18	0.65 (0.24, 1.80)	.41	0.65 (0.22, 1.95)	.44
Psychoses	93	7	0.71 (0.31, 1.60)	.41	0.71 (0.31, 1.59)	.40	42	7	0.65 (0.12, 3.54)	.61	0.61 (0.13, 2.89)	.53
Pulmonary circulation disorders	100	8	1.26 (0.65, 2.47)	.49	1.34 (0.70, 2.59)	.38	45	8	2.11 (0.59, 7.54)	.25	1.98 (0.56, 6.96)	.29
Dementia	35	3	3.10 (1.38, 6.96)	.006	3.22 (1.47, 7.06)	.003	13	2	1.78 (0.61, 5.22)	.29	1.47 (0.46, 4.69)	.51
BMI (kg/m^2) (mean, std. dev.)	29.3	8.9	1.03 (1.02, 1.05)	<.001	1.03 (1.02, 1.05)	<.001	29.1	8.5	1.04 (0.99, 1.08)	.10	1.03 (0.99, 1.07)	.16
Goodness-of-link test of squared predicted value				.07		.16				.44		.56
Hosmer-Lemeshow goodness-of-fit decile test				.86		.88				.68		.44

* Vitamin D dose was rank-ordered as follows: Calcitriol > 4001+ IU D3 > 2001-4000 IU D3 > 1001-2000 IU D3 > D2 > 1-1000 IU D3/Multivitamin > No Vitamin D.
† Months since most recent calcifediol level was defined as the number of days between their most recent calcifediol level and 14 days before their first COVID-19 test order, divided by (365/12).
ξ Stata did not produce a reliable coefficient estimate for this covariate because 0 of the persons in the subgroup tested positive for COVID-19, so we omitted the covariate as indicated.

© 2021 Meltzer DO et al. *JAMA Network Open.*

eFigure. COVID-19 Test Positivity Rate by Race and by Most Recent Vitamin D Levels Before COVID-19 Tests

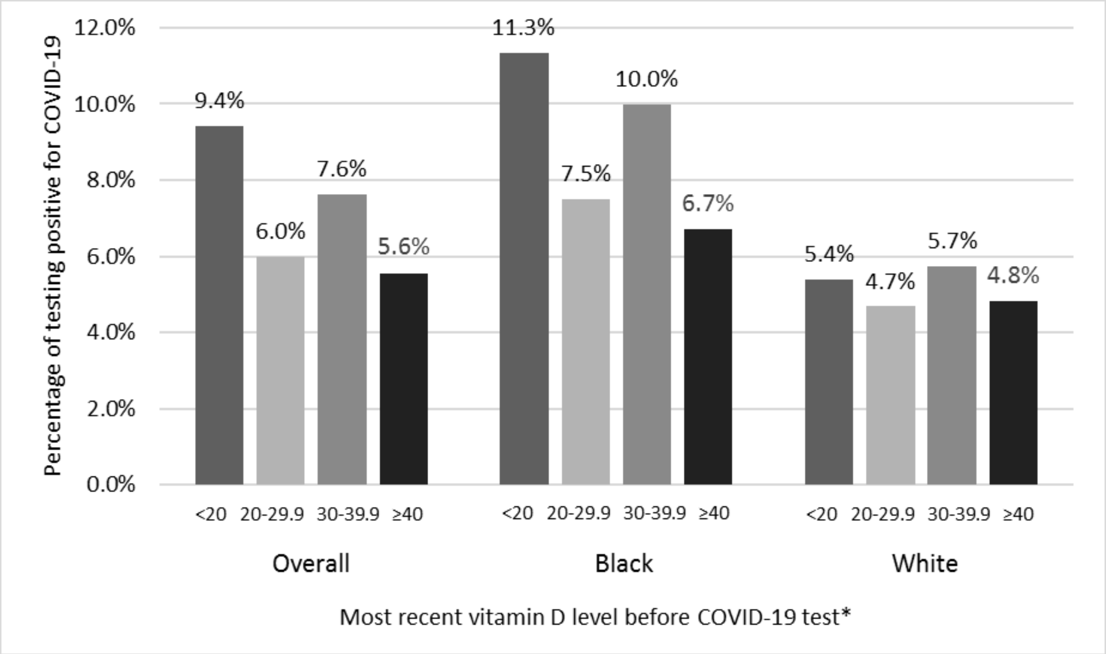

* Defined as a person's most recent calcifediol level ordered from 14 to 365 days before the person's first COVID-19 test order.

© 2021 Meltzer DO et al. *JAMA Network Open.*

eAppendix. Supplemental Methods

Chronic condition indicators. Each chronic condition indicator was set equal to 1 if the person had at least one of a list of ICD-10-CM diagnosis codes included on an administrative/billing record with a discharge date from 14 to 730 days before the person's first COVID-19 test order. The lists of codes used for the indicators was defined using the HCUP Elixhauser Comorbidity Software.[20] For each condition, we used all ICD-10-CM codes listed within categories in the Elixhauser file Comorb_ICD10CM_Format_v2021-1.sas, which is entitled Creation of Format Library for Comorbidity Groups, ICD-10-CM Comorbidity Software, Version 2021.1. The specific ICD-10-CM codes can be found by finding the following category variable names (listed in all caps) in the SAS file named above. Hypertension: CHFHTN_CX, CHFHTN_CXRENLFL_SEV, HTN_CX, HTN_CXRENLFL_SEV, HTN_UNCX. Comorbidities with Immunosuppression: AIDS, ARTH, CANCER_LEUK, CANCER_LYMPH, CANCER_METS, CANCER_SOLID. Diabetes: DIAB_CX, DIAB_UNCX. Renal failure: CHFHTN_CXRENLFL_SEV, HTN_CXRENLFL_SEV, RENLFL_MOD, RENLFL_SEV. Chronic pulmonary disease: LUNG_CHRONIC. Depression: DEPRESS. Liver disease: ALCOHOLLIVER_MLD, LIVER_MLD, LIVER_SEV. Psychoses: DRUG_ABUSEPSYCHOSES, PSYCHOSES. Pulmonary circulation disorders: PULMCIRC. Dementia: DEMENTIA.

© 2021 Meltzer DO et al. *JAMA Network Open.*
